# MPV17 does not control cancer cell proliferation

**DOI:** 10.1371/journal.pone.0229834

**Published:** 2020-03-10

**Authors:** Morgane Canonne, Anaïs Wanet, Thuy Truong An Nguyen, Alexis Khelfi, Sophie Ayama-Canden, Martine Van Steenbrugge, Antoine Fattaccioli, Etienne Sokal, Mustapha Najimi, Thierry Arnould, Patricia Renard

**Affiliations:** 1 Laboratory of Biochemistry and Cell Biology (URBC), NAmur Research Institute for LIfe Sciences (NARILIS), University of Namur (UNamur), Namur, Belgium; 2 Laboratory of Pediatric Hepatology and Cell Therapy, Institut de Recherche Expérimentale et Clinique (IREC), Université Catholique de Louvain, Brussels, Belgium; National Institute of Environmental Health Sciences (NIEHS), UNITED STATES

## Abstract

MPV17 is described as a mitochondrial inner membrane channel. Although its function remains elusive, mutations in the *MPV17* gene result in hepato-cerebral mitochondrial DNA depletion syndrome in humans. In this study, we show that *MPV17* silencing does not induce depletion in mitochondrial DNA content in cancer cells. We also show that MPV17 does not control cancer cell proliferation despite the fact that we initially observed a reduced proliferation rate in five MPV17-silenced cancer cell lines with two different shRNAs. However, shRNA-mediated *MPV17* knockdown performed in this work provided misguiding results regarding the resulting proliferation phenotype and only a rescue experiment was able to shed definitive light on the implication of MPV17 in cancer cell proliferation. Our results therefore emphasize the caution that is required when scientific conclusions are drawn from a work based on lentiviral vector-based gene silencing and clearly demonstrate the need to systematically perform a rescue experiment in order to ascertain the specific nature of the experimental results.

## Introduction

MPV17 is a functionally elusive protein localized in the inner membrane of mitochondrion and for which the encoding gene is located on chromosome 2p23-21 [1] [2] [3] [4]. MPV17 loss-of-function causes a rare autosomal recessive mitochondrial disorder called Mitochondrial DNA Depletion Syndrome (MDDS) marked by a highly reduced mitochondrial DNA (mtDNA) copy number in affected tissues.

To date, there are 100 known individuals affected by one of the 48 described *MPV17* pathogenic variants (approximately half of which are missense) [5]. The vast majority of these patients (96%) suffer from the hepato-cerebral form of MDDS and exhibit a severe mtDNA depletion in the liver (60–99% reduction). This is correlated with a decreased activity of respiratory chain complexes. The onset of the disease takes place early in life (neonatal period/infancy) and condemn the affected individual to a premature death due to liver dysfunction progressing into liver failure. The remaining 4% of the patients suffer from a late-onset encephalomyopathic disease with mild or no liver involvement [5].

Although the current knowledge about MPV17 and its homologs have been recently reviewed [6], the function of MPV17 is still obscure. There are evidence supporting that MPV17 might be a channel with stress-dependent gating properties [7] [8] involved in nucleotides homeostasis [9] [3] [10] [11] [12].

In a previous work, we found an increased expression of *MPV17* during stem cells hepatogenic differentiation while performing a transcriptomic analysis in order to characterize the mitochondrial biogenesis in this process ([13], data deposited in NCBI's Gene Expression Omnibus through GEO Series accession number GSE75184). Unexpectedly, we observed that while *MPV17* silencing had no impact on hepatogenic differentiation, it significantly reduced the proliferation of expanding Bone Marrow Mesenchymal Stem Cells (MSC) and Umbilical Cord-MSC from different donors, suggesting a role of MPV17 in cell proliferation (unpublished data). This is in accordance with the work of Choi and colleagues who showed that *MPV17* knockdown reduces the proliferation of NSC34 cells, a mouse motor neuronal cell line [14]. As MPV17 has been implicated in stress response [15] [16] and has been described as a channel with stress-dependent gating properties (oxidative and pH stress,…) [7], we then wondered whether MPV17 could have a role in the proliferation of cancer cells or not, as they inherently experience oxidative and metabolic stress.

In order to explore the putative role of MPV17 in the control of cancer cell proliferation, we used a loss-of-function approach. Gene silencing and/or overexpression is generally the first approach in order to investigate gene expression/function and lentiviruses are now widely used to deliver transgenes that integrate into the host genome for gene expression tampering. In this study, while demonstrating that MPV17 does not control neither cell proliferation nor mtDNA content in cancer cells, our experimental results illustrate and emphasize the importance of carrying out a rescue experiment when working with shRNA-mediated knockdown.

## Results

### *MPV17* is overexpressed in several tumours

**Fig 1:**
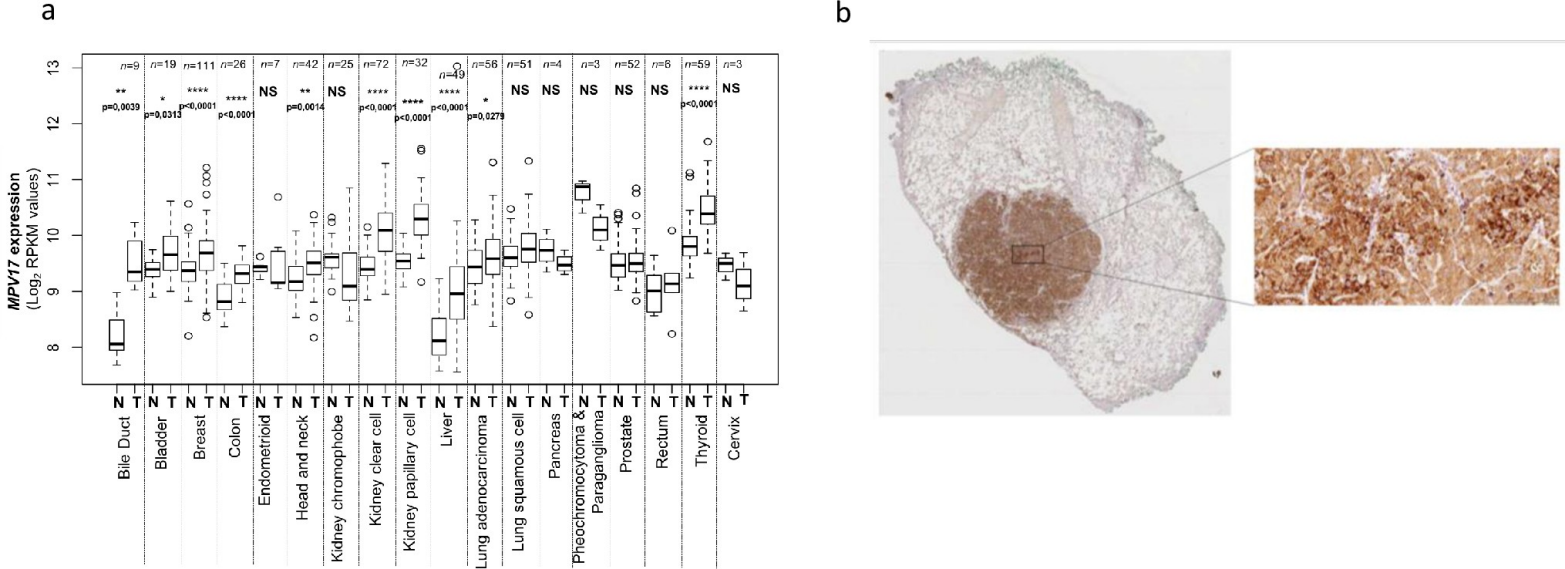
Analysis of MPV17 expression in cancer tissues. The Cancer Genome Atlas (TCGA), a public platform allowing the analysis of gene expression data sets generated by RNA sequencing (http://cancergenome.nih.gov), has been used to determine the expression level of *MPV17* in various tumour tissues (T) *versus* healthy tissues (N). P values were calculated with the two-tailed Wilcoxon signed rank Test (⍺ = 5%; *: p<0.05; **: p<0.01; ***: p<0.001; ****: p<0.0001; NS: not significant) (a). Detection of MPV17 by immunohistochemistry in a paraffin-embedded biopsy of a liver adenocarcinoma. A strong signal for MPV17 is associated with the tumour, while the abundance of the protein is low in adjacent normal tissue (b).

Taking advantage of The Genome Cancer Atlas (TGCA) database, we show that the abundance of *MPV17* transcript is significantly higher in tumours of 10 different tissues, including liver, bile duct, and colon (Fig 1A). This is confirmed at the protein level by immunohistochemistry staining performed on liver tumour biopsies from patients with adenocarcinoma (Fig 1B).

### *MPV17* silencing is robustly associated with a decreased proliferation rate in different cancer cell lines

**Fig 2:**
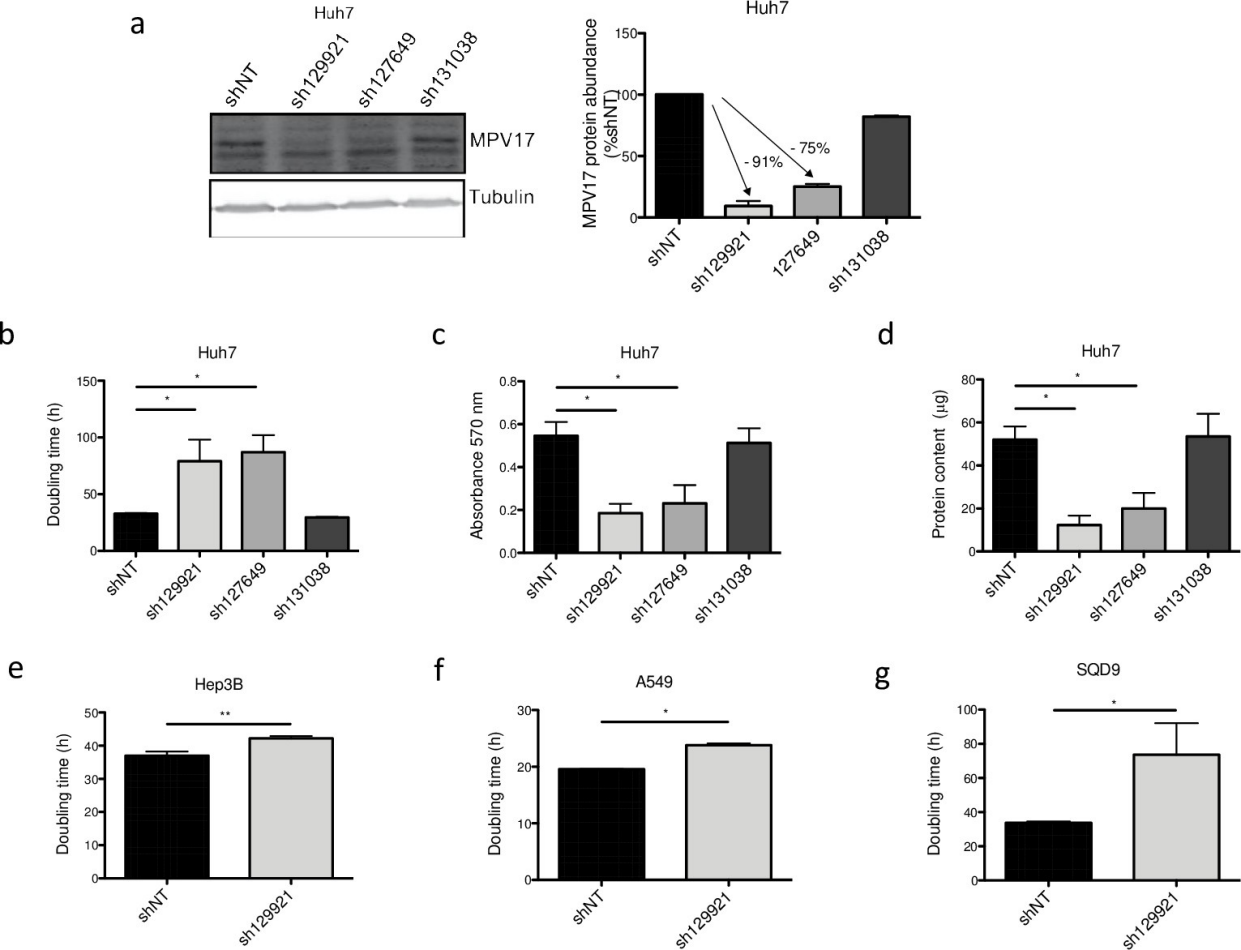
Effect of shRNA-mediated *MPV17* knockdown on the proliferation of cancer cell lines. Huh7 cells were transduced with non-target shRNA lentiviral vectors (shNT) or with shRNA lentiviral vectors targeting *MPV17* expression (sh129921, sh127649, sh131038). Hep3B, A549 and SQD9 cells were transduced with lentiviral shNT-encoding vectors or with sh129921-containing vectors. Cells were selected for 6 days with puromycin (2.5 μg/mL). Cells were seeded at 8×10^3^ cells/cm^2^ (a, b, c, d, Huh7; g, SQD9), 5×10^3^ cells/cm^2^ (e, Hep3B) and 2.7×10^3^ cells/cm^2^ (f, A549) and grown for 4 days. MPV17 protein abundance was assessed by western blot analysis (a, Huh7). A representative western blot of 3 independent biological replicates (2 for sh131038) is shown (left) along with the western blot quantification of all the biological replicates (quantification with Image J software, data expressed as relative protein abundance to cells transduced with shNT-encoding vectors, right). Proliferation was then assessed by manual counting to calculate the doubling time (b, Huh7; e, Hep3B; f, A549; g, SQD9), by MTT assay (c, Huh7) and by the total protein content (d, Huh7). Data are presented as mean ± S.E.M of 3 independent biological replicates (2 for sh131038). P values were calculated with the one-tailed Mann-Whitney Test (⍺ = 5%; *: p<0.05; **: p<0.01; ***: p<0.001). Full blots are presented in S6 Fig.

In this study, we first assessed the effects of three commercially available shRNAs targeting *MPV17* mRNA: sh129921, sh127649 and sh131038. Using western blot analysis, the efficiency of gene silencing was evaluated by assessing the abundance of MPV17 in Huh7 cells transduced with these three shRNA-encoding lentiviral vectors. Both sh129921 and sh127649 led to an efficient knockdown of the gene while sh131038 did not efficiently induce *MPV17* silencing (Fig 2A).

We then demonstrated that Huh7 cells silenced for *MPV17* with sh129921 and sh127649 displayed a severely decreased proliferation rate, as quantified by three different proliferation assays, namely the doubling time (Fig 2B), the MTT assay (Fig 2C), and the total protein content (Fig 2D). The reduced cell proliferation phenotype was correlated with *MPV17* knockdown efficiency as no decreased proliferation rate was observed in Huh7 cells transduced with the vector encoding sh131038, the only shRNA that turned out to be inefficient in the knockdown induction.

To discard the possibility of a putative cell type or cancer type-specific phenotype, we next assessed the impact of sh129921 on the proliferation of two other human hepatoma cell lines, Hep3B and HepG2 cells, and two non-liver cancer cell lines, A549 cells, derived from a human pulmonary adenocarcinoma and SQD9 cells, a human squamous cell carcinoma cell line. Interestingly, Hep3B (Fig 2E), A549 (Fig 2F), SQD9 (Fig 2G) and HepG2 (data not shown) cells transduced with sh129921-encoding vector also displayed a reduced proliferation phenotype.

### *MPV17* silencing is not associated with mtDNA copy number depletion

**Fig 3:**
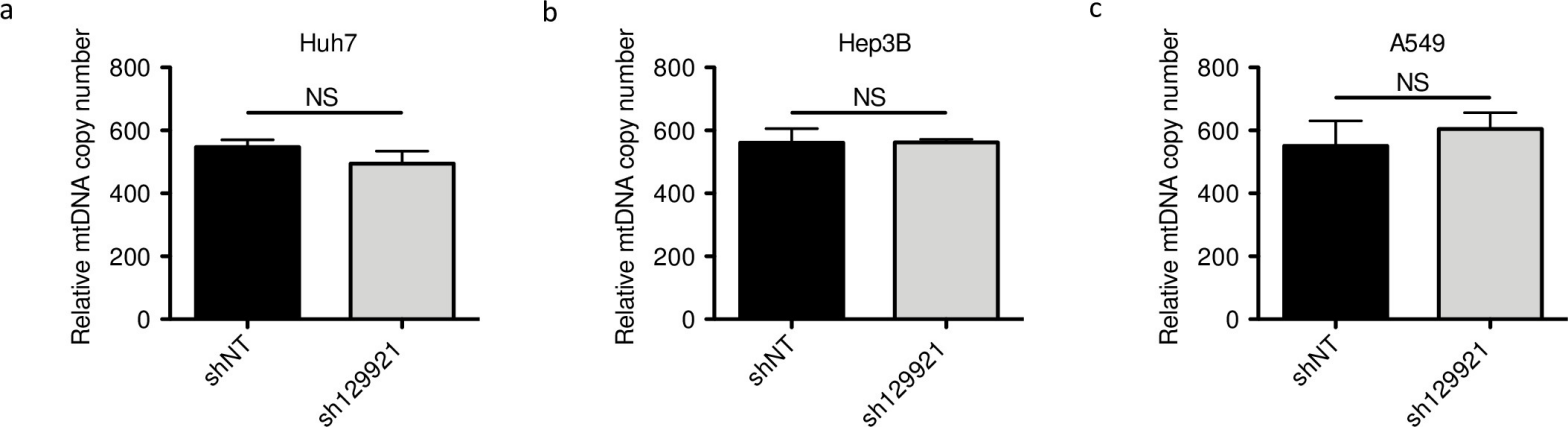
Assessment of mtDNA content in *MPV17*-silenced cancer cell lines. Huh7 (a), Hep3B (b) and A549 (c) cells were transduced with non-target shRNA lentiviral vectors (shNT) or with sh129921-containing vectors. Transduced cells were selected for 6 days with puromycin (2.5 μg/mL). DNA was then extracted and mtDNA content was assessed by qPCR using NADH dehydrogenase 2 as a specific marker of mtDNA content and beclin for normalization with nuclear DNA. Results are presented as means ± S.E.M of 3 independent biological replicates and are expressed in relative copy number to the nuclear DNA. P values were calculated with the one-tailed Mann-Whitney Test (⍺ = 5%; NS; *: p<0.05; **: p<0.01; ***: p<0.001).

As MPV17 deficiency is associated with MDDS, we assessed whether *MPV17* silencing was accompanied by depletion in the mtDNA content or not, possibly accounting for the associated decreased proliferation rate. However, *MPV17* silencing did not lead to a reduction of mtDNA content in any of the tested cancer cell lines (Fig 3). This result is in agreement with the work of Dalla Rosa and collaborators who showed that proliferating fibroblasts from *MPV17*-deficient patients do not display any reduced mtDNA content [9]. It is also in accordance with the recent work of Alonzo and collaborators who did not find any reduction of mtDNA content in *MPV17*-silenced HeLa cells [12].

### The decreased proliferation rate in *MPV17*-silenced cells is associated with a decrease in the abundance of ATF4

**Fig 4:**
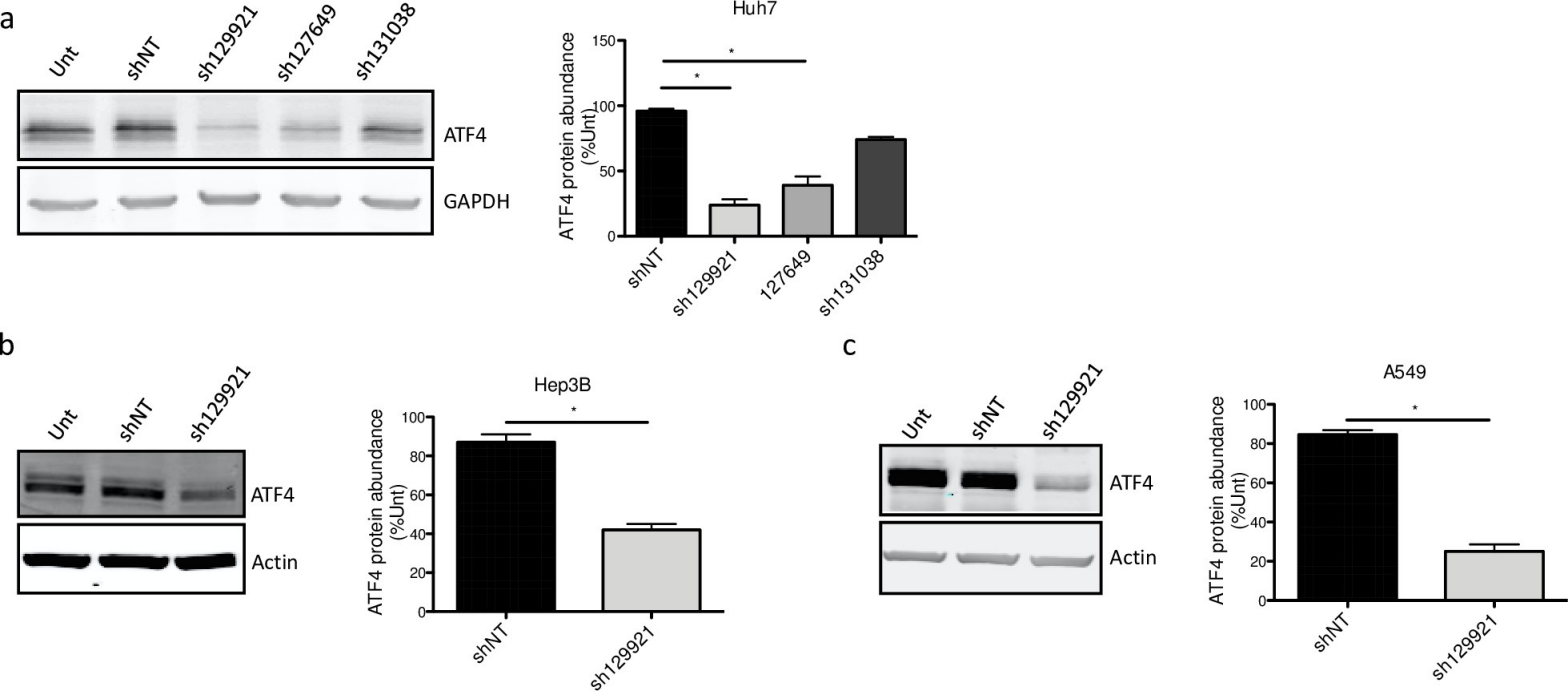
ATF4 protein abundance following *MPV17* knockdown in Huh7, Hep3B and A549 cells. Huh7 cells (a) were transduced with non-target shRNA lentiviral vectors (shNT) or with shRNA lentiviral vectors targeting *MPV17* expression (sh129921, sh127649, sh131038). Hep3B (b) and A549 (c) cells were transduced with lentiviral shNT-encoding vectors or with sh129921-containing vectors. Cells were selected for 6 days with puromycin (2.5 μg/mL) before assessing ATF4 protein abundance by western blot analysis. For each cell line, a representative western blot of 3 (2 for sh131038) independent biological replicates is shown (left) along with the western blot quantification of all the biological replicates (quantification with Image J software, data expressed as relative protein abundance to untranduced (unt) cells, right). P values were calculated with the one-tailed Mann-Whitney Test (⍺ = 5%; *: p<0.05; **: p<0.01; ***: p<0.001). Full blots are presented in S6 Fig.

In order to determine the molecular mechanisms underlying the decreased proliferation rate in *MPV17*-silenced cancer cells, a transcriptomic analysis was performed. The RNA sequencing analysis was performed on Huh7 cells transduced with sh129921 or shNT-encoding vectors. Among the differentially expressed genes, we focused on transcriptional regulators potentially responsible for a reduced proliferation capacity. The activating transcription factor 4 (ATF4) frequently upregulated in cancer cells [17], was downregulated in *MPV17*-silenced cells (Z-score = -2,792; *P* = 1,38E-14). It has been shown that ATF4 not only up-regulates the expression of genes encoding actors implicated in amino acid import and metabolism [18] [19] [20] but also promotes, indirectly, purine synthesis [21], two essential aspects for cell proliferation. Based on this knowledge, ATF4 was an attractive candidate in the attempt to elucidate the molecular mechanisms underlying the reduced cell proliferation. The reduction of *ATF4* transcript abundance was confirmed at the protein level in sh129921 and sh127649-encoding vector transduced Huh7 cells (Fig 4A) as well as in sh129921-encoding vector transduced Hep3B (Fig 4B) and A549 (Fig 4C) cells, when compared with control cells transduced with shNT-containing vector.

Altogether, these results seem to strongly support an involvement of MPV17 in cancer cell proliferation as *MPV17* silencing was consistently accompanied by a reduction of both cell proliferation rate and ATF4 protein abundance.

### *MPV17* silencing is not always associated with a reduced proliferation phenotype

**Fig 5:**
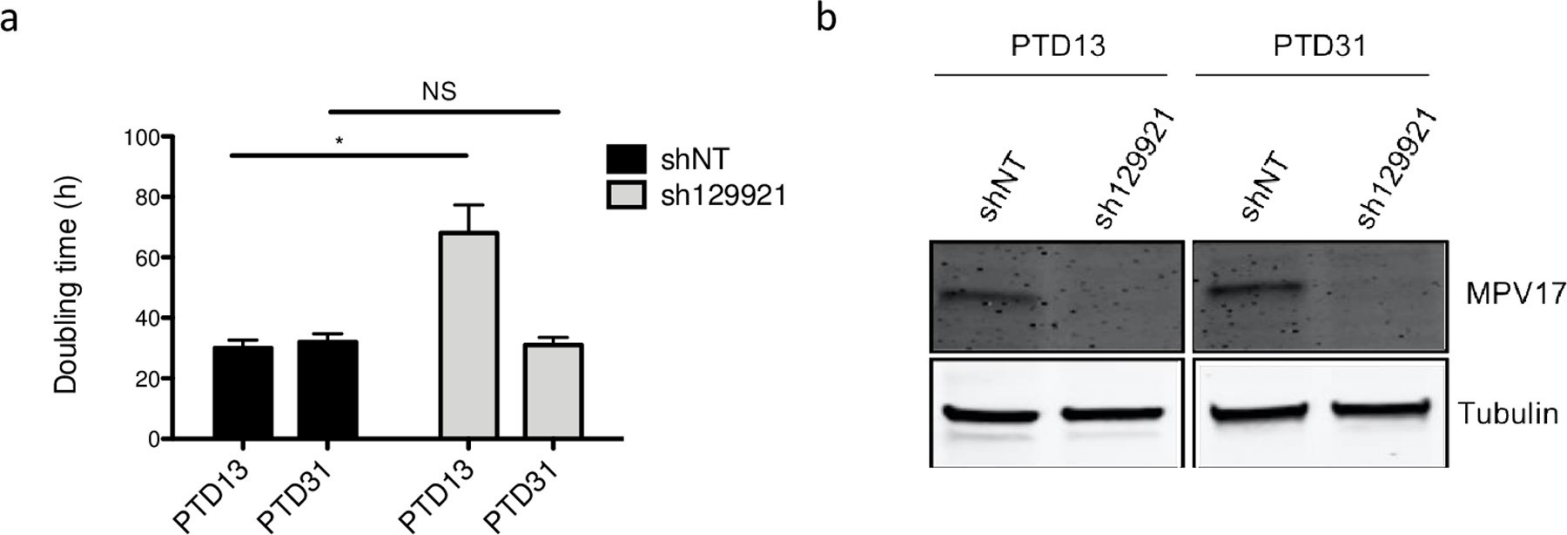
Evolution of Huh7 proliferation rate and MPV17 protein abundance in sh129921-mediated *MPV17* knockdown. Huh7 cells were transduced with non-target shRNA lentiviral vectors (shNT) or with sh129921-containing vectors. Transduced cells were selected for 6 days with puromycin (2.5 μg/mL). At 13 and 31 days after the transduction (PTD: post-transduction day), cells were seeded at 8×10^3^ cells/cm^2^ and grown for 4 days. Proliferation was then assessed by manual counting to calculate the doubling time (a) and MPV17 protein abundance was analysed by western blot (b). P values were calculated with the one-tailed Mann-Whitney Test (⍺ = 5%; NS; *: p<0.05; **: p<0.01; ***: p<0.001). n = 3. Full blots are presented in S6 Fig.

Pursuing our analysis further, we observed that the sh129921-encoding vector transduced Huh7 cells were able to adapt along the passages and progressively restored the proliferation rate (Fig 5A), although MPV17 protein abundance was still strongly reduced (Fig 5B).

**Fig 6:**
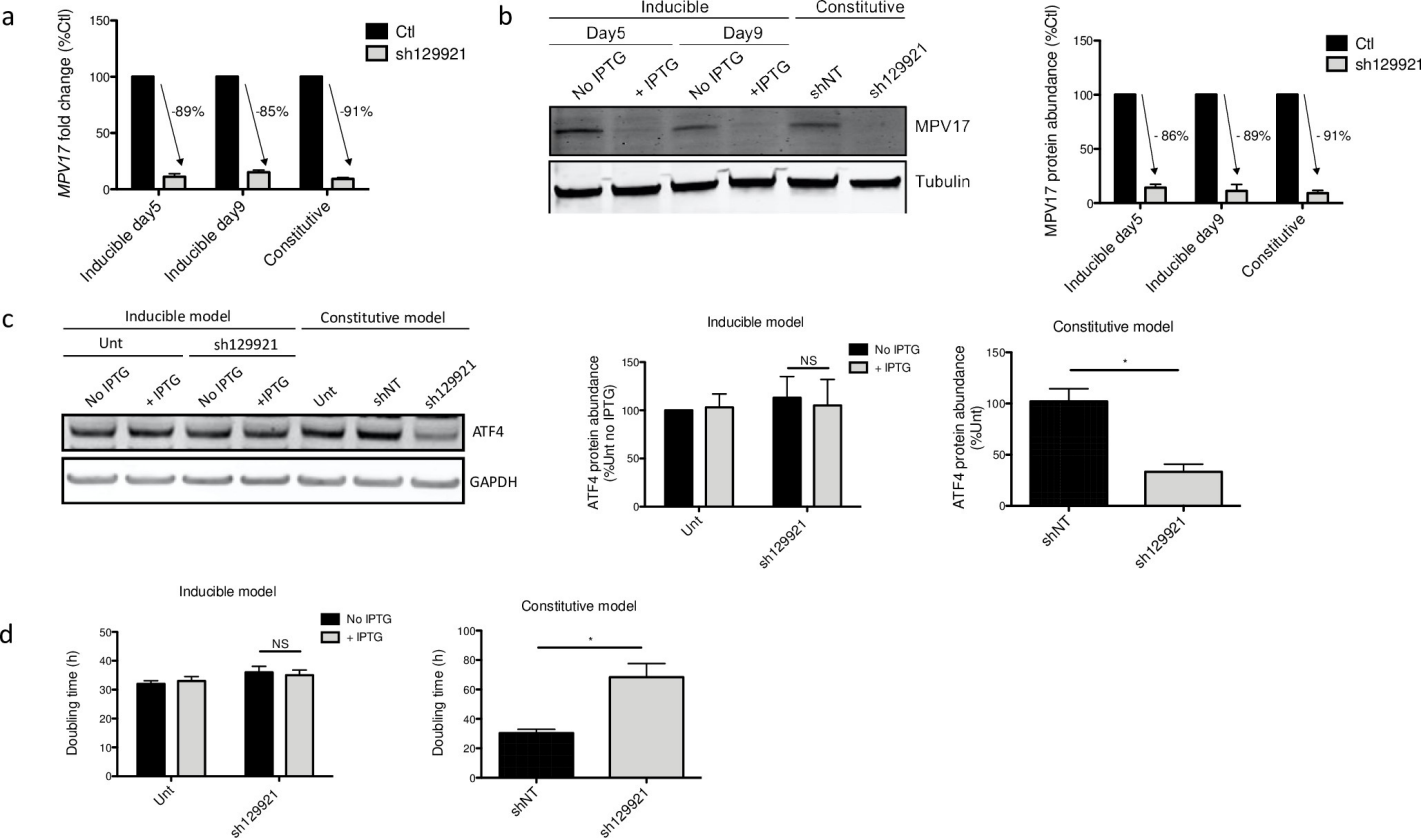
MPV17 and ATF4 abundances and Huh7 cells proliferation rate following inducible sh129921-mediated *MPV17* knockdown. Huh7 cells were transduced, or not (Unt), with inducible sh129921 lentiviral vector. Transduced cells were selected for 6 days with puromycin (2.5 μg/mL). Cells were then incubated for 5 days in the presence of 0.1 mM of IPTG to induce *MPV17* silencing. Cells were seeded at 8×10^3^ cells/cm^2^ and grown for 4 days in daily renewed medium containing IPTG. RNA was extracted and RT-qPCR was performed to assess *MPV17* transcript level (data expressed relatively to respective control (Ctl), n = 3) (a). MPV17 (b) and ATF4 (c) protein abundances were also assessed by western blot analysis after 5 days (MPV17) and 9 days (MPV17; ATF4) of IPTG induction. For each protein, a representative western blot of 3 independent biological replicates is shown (left) along with the western blot quantification of all the biological replicates (quantification with Image J software, data expressed as relative protein abundance to respective controls (Ctl) for MPV17 and untransduced (unt) cells for ATF4, right). Proliferation was then assessed by manual counting to calculate the doubling time (d). As a comparison, we performed at the same time and on the same cells a similar experiment with the constitutive expression of sh129921 (mediating *MPV17* knockdown) as described in S1 Fig. P values were calculated with the one-tailed Mann-Whitney Test (⍺ = 5%; *: p<0.05; **: p<0.01; ***: p<0.001). Full blots are presented in S6 Fig. n = 3.

As we observed a recovery of the decreased proliferation rate over time, we therefore aimed at generating an IPTG-inducible sh129921 model in Huh7 cells. Strikingly, while we observed a strong *MPV17* knockdown in this inducible expression model, with a silencing efficiency comparable to the one observed in the constitutive silencing model (Fig 6A and 6B), the cell proliferation rate was unchanged (Fig 6D). Moreover, ATF4 did not display a decreased protein abundance (Fig 6C).

This absence of effect of *MPV17* silencing on the cell proliferation rate in the inducible expression system could have different origins. First, the IPTG molecule itself could have an unexpected effect on Huh7 cells proliferation, even though IPTG is not known to be metabolized [22]. Second, the reduced proliferation phenotype in the inducible expression system might need more time to settle down. Indeed, in the constitutive system, *MPV17* is silenced for a total of 9 days before assessing the cell proliferation, as opposed to only 5 days in the inducible model (see “Materials and Methods”, S1 Fig). A third difference between the two approaches is that in order to ensure a proper knockdown of *MPV17* in the inducible model, the culture medium was changed every day in order to renew the IPTG, as opposed to every two or three days in the constitutive silencing model. One could thus hypothesize that this daily medium renewal could prevent the settling of the reduced proliferation phenotype by discontinuing the putative intercellular communication. These three hypotheses were tested but failed to explain the different phenotypical outcomes observed for the constitutive or inducible expression models (see S2 and S3 Figs).

**Fig 7:**
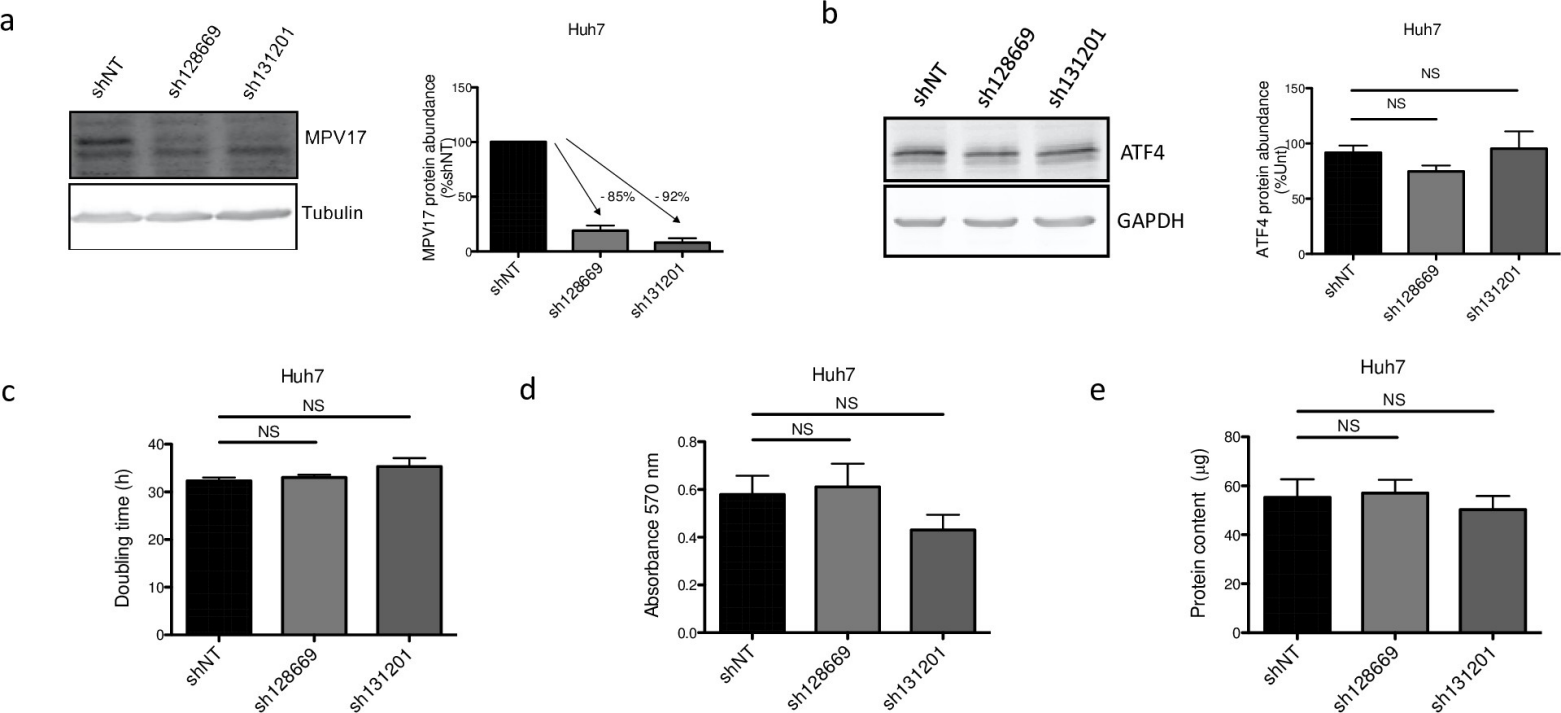
Effect of *MPV17*-targeting sh128669 and sh131201 on Huh7 cells proliferation, MPV17 and ATF4 protein abundances. Cells were transduced with non-target shRNA lentiviral vectors (shNT) or with shRNA lentiviral vectors targeting *MPV17* expression (sh128669 and sh131201). Transduced cells were selected for 6 days with puromycin (2.5 μg/mL). Cells were seeded at 8×10^3^ cells/cm^2^ and grown for 4 days. The abundance of MPV17 (a) and ATF4 (b) proteins was assessed by western blot analysis. For each protein, a representative western blot analysis of 3 independent biological replicates is shown (left) along with the western blots quantification of all the biological replicates (quantification with Image J software, data expressed as relative protein abundance to cells transduced with shNT-encoding vectors (a, MPV17) or untranduced (unt) cells (b, ATF4), right). Proliferation was then assessed by manual counting to calculate the doubling time (c), by MTT assay (d) and by the total protein content (e) and data are presented as mean ± S.E.M (3 biological replicates). P values were calculated with the one-tailed Mann-Whitney Test (⍺ = 5%; NS; *: p<0.05; **: p<0.01; ***: p<0.001). Full blots are presented in S6 Fig.

These considerations led us to evaluate on Huh7 cells the effect of two additional commercially available shRNAs, sh128669 and sh131201, targeting different regions of the *MPV17* transcript (see S4 Fig). Both shRNAs strongly reduce the abundance of MPV17 protein (Fig 7A) but no significant effect on the proliferation rate was observed (Fig 7C, 7D and 7E). Also, the abundance of ATF4 was not affected by *MPV17* knockdown mediated by either sh128669 or sh131201 (Fig 7B). Thus, ATF4 reduced abundance correlates with the proliferation rate but the putative link between the reduced proliferation phenotype (and therefore ATF4) and MPV17 remains to be established.

In conclusion, we found that sh129921, sh127649, sh128669 and sh131201 all led to a strong *MPV17* knockdown while the resulting proliferation rates were highly variable. This lack of consistency led us to suspect a putative involvement of *MPV17* isoforms that would be differentially affected according to the shRNA used.

Therefore, we next interrogated the Genotype-Tissue Expression (GTEx) Consortium (2008, NIH) portal, that inventories the impact of genetic variations on gene expression within major tissues in the human body from *post mortem* donors. The GTEx Portal proposes 22 *MPV17* isoforms, including two major ones i.e. a short predominant isoform and a long one (see S5 Fig). This long isoform referenced in RefSeq as NM002437.5 encodes the MPV17 protein that we detected on western blot while the translation of all the other *MPV17* isoforms is not experimentally demonstrated. However, among the 22 isoforms, we observe that all the transcripts targeted by sh129921 (1, 2, 3, 12, 13, 14, 15, 16 and 18) are also targeted by either sh128669 (15, 16) or sh131201 (1, 18) or both (2, 3, 12, 13, 14). The same kind of observation stands for the transcripts targeted by sh127649 (1, 2, 3, 5, 12, 13, 14, 15, 16, 18, 19, 21), providing no clear explanation about the opposite proliferation phenotypes observed for each pair of shRNAs (sh129921/sh127649 versus sh128669/131201) (see S5 Fig).

Altogether, the absence of effect of the inducible sh129921 on the cell proliferation rate, combined with the observation that, at least, two different shRNAs targeting *MPV17* (sh131201 and sh128669) have no effect on the proliferation of Huh7 cells, despite a strong decrease in the MPV17 protein abundance, suggest that the decreased proliferation rate of transduced cells observed for two shRNAs (sh129921 and sh127649) is not related to a reduced MPV17 protein abundance. In an attempt to shed light on this question, we eventually performed a rescue experiment.

#### Rescuing MPV17 does not restore the proliferation phenotype

As Huh7 cells could neither be efficiently transfected nor transduced with the *MPV17*-expressing vector, HepG2 cells were used for the rescue attempt. Indeed, as mentioned before, we also observed the reduced proliferation rate in this hepatoma cell line silenced for *MPV17*. This cell line turned out to be the most receptive to the lentiviral *MPV17*-expressing vector. Since a rescue experiment consists in re-introducing the shRNA-mediated silenced mRNA, it requires the use of a shRNA targeting the 3′UTR of the targeted mRNA. Thus, the reintroduced transcript that lacks 3′UTR is not affected by the shRNA.

We decided to perform MPV17 overexpression before inducing the constitutive knockdown mediated by sh129921. The justification of this chronology resides in the observation that, on the contrary to shNT-encoding vector transduced cells, sh129921-encoding vector transduced cells were not able to stand a second round of transduction due to cell death. This suggests that *MPV17*-silenced cells are distressed, in accordance with their decreased proliferation rate. This aspect is therefore not compatible with a short-term assessment of cell proliferation.

**Fig 8:**
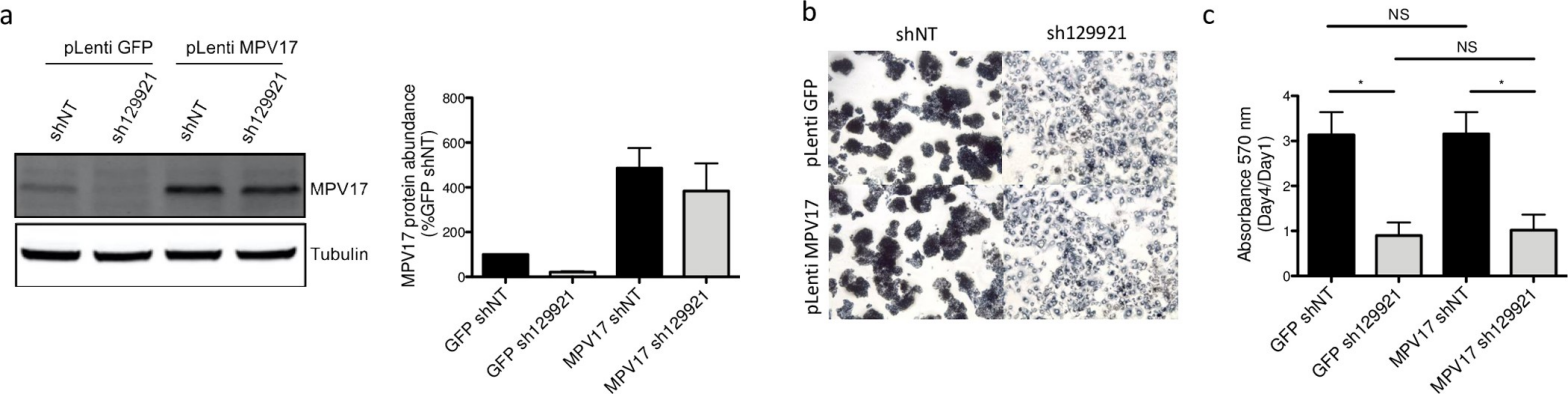
Effect of MPV17 rescue on the proliferation of *MPV17*-silenced HepG2 cells. Cells were first transduced with pLenti GFP or pLenti MPV17 and selected for 6 days with puromycin (2.5 μg/mL). Cells were then transduced with shNT or sh129921-encoding lentiviral vectors and let to recover for 5 days to allow *MPV17* silencing. Cells were then seeded at 1.5×10^4^ cells/cm^2^ and grown for 4 days before assessing MPV17 protein abundance (a). A representative western blot analysis of 3 independent biological replicates is shown (left) along with the western blots quantification of all the biological replicates (quantification with Image J software, data expressed as relative protein abundance to cells transduced with shNT and GFP-encoding vectors, right). Cell proliferation assessment by MTT assay was also performed at day 1 and day 4. The ratio day 4/day 1 is then calculated in order to correct any putative seeding differences that could mask or mislead to a partial phenotype rescue (c). Micrographies were taken at the phase contrast microscope before adding the lysis buffer for the MTT assay (b). Data are presented as mean ± S.E.M (3 biological replicates). P values were calculated with the one-tailed Mann-Whitney Test (⍺ = 5%; NS; *: p<0.05; **: p<0.01; ***: p<0.001). Full blots are presented in S6 Fig.

*MPV17* knockdown was properly induced in the overexpression control. Indeed, cells double transduced with pLenti GFP, as a control, and then with pLKO.1 sh129921, displayed a strong reduction of MPV17 abundance accompanied by the decreased proliferation rate (Fig 8A, 8B and 8C). As expected, the reduced proliferation phenotype was absent in pLenti GFP and pLKO.1 shNT double-transduced cells (Fig 8B and 8C). Cells double-transduced with pLenti MPV17 and pLKO.1 shNT suitably overexpressed MPV17 (Fig 8A). It is interesting to emphasize the fact that MPV17 overexpression, on its own, has no significant effect on cell proliferation (Fig 8B and 8C). This observation can be reconciled with the idea that MPV17 is described as a channel. Thus, the qualitative state of MPV17 (open/closed) would be more relevant than its quantitative state. Finally, cells double-transduced with pLenti MPV17 and pLKO.1 sh129921 overexpressed MPV17 but lost the expression of the endogenous protein (Fig 8A). Remarkably, these cells exhibited the reduced proliferation phenotype (Fig 8B and 8C).

## Discussion

In this work we studied the putative role of MPV17 in cancer cell proliferation. While *MPV17* silencing did not lead to depletion of mtDNA content in the tested cancer cell lines, in accordance with literature [9] [12], the resulting proliferation phenotypes associated with *MPV17* shRNA-mediated knockdown were unsettling and inconclusive, leading us to perform a rescue experiment that eventually excluded a role of MPV17 in cancer cell proliferation. The intriguing and somewhat misleading results we obtained during this investigation clearly demonstrate the need to systematically perform a rescue experiment in order to ascertain the specific nature of the experimental results.

In this work, we tested five shRNAs targeting *MPV17*. The highly variable phenotypical outcomes in Huh7 cells led us to consider a putative contribution of *MPV17* transcript isoforms or the possible existence of an off-target effect. Despite the fact that we did not find any correlation between the shRNA-targeted *MPV17* transcript isoforms and the proliferation phenotype, it is not sufficient to preclude this hypothesis as there is no guaranty that the *MPV17* transcript isoforms spectrum described in GTEx portal in human healthy liver is conserved in our model.

It is also interesting to note that the predictive alignment (BLAST, ncbi) of sh129921 and sh127649 on the human transcripts did not highlight the putative existence of a shared off-target mRNA. Again, this observation is however not sufficient to exclude a putative off-target effect as it could be taking place through separate effectors for each shRNA.

However, the most intriguing observation in this work was that the same shRNA, expressed either constitutively or induced by IPTG, resulted in different effects on cell proliferation. The emergence of the inducible lentiviral vectors, which enable a reversible and fine tuning of gene knockdown, allows further characterization of gene function, a better ease in studying genes for which knockdown ends up being too deleterious or lethal for the cell, as well as timeframe selection in a developmental process [23]. In this study, we resorted to this tool to overcome an apparent progressive compensation/adjustment of the cells following *MPV17* knockdown. The transient nature of the inducible knockdown would theoretically prevent this compensation to settle. However, we unexpectedly observed that the constitutive and inducible approaches led to different phenotypical outcomes, despite using the same shRNA sequence (sh129921). An explanation to this observation could reside in the fact that the shRNA is expressed at a higher level in the constitutive model. Indeed, it is recommended by Sigma Aldrich to ensure a minimum of 70% knockdown in a constitutive model, before switching to an inducible one. This recommendation reflects the risk of obtaining a less potent gene silencing with an inducible vector when compared to a constitutive one due to a lower shRNA expression. In our experimental conditions, the observation that MPV17 knockdown at the protein level was quite comparable in both expression models does not preclude a higher expression of the shRNA in the constitutive expression system, which could either increase the risk of off-targeting or favour a better targeting of some non coding *MPV17* transcript isoforms.

While most researchers are confident in their interpretation of a particular gene function when a consistent phenotype is observed with at least two different shRNA sequences targeting a transcript, we and others experienced that this is not sufficient and that a rescue experiment is an all-encompassing insurance of the veracity of the scientific conclusions [24]. However, as properly reported by Peretz and collaborators, “Rescue experiments are a good way to ensure specificity and are being added to an increasing number of studies, although, based on a survey of scientific literature, this is probably limited to less than 0.1% of studies” [24]. Our work stresses the imperative necessity to perform a rescue experiment in each study carrying out shRNA-mediated gene knockdown. Nevertheless, the benefit of a rescue experiment often matches its technical complexity. In terms of limitations while performing a rescue experiment, we can mention that the re-expression of the silenced gene, generally at a higher level than the normal endogenous one, might, in itself, lead to undesired effects [24]. In addition, the random integration of the vector can also lead to additional off-target effects. Although the rescue experiment should undeniably be an indispensable control in every experiment based on shRNA-induced knockdown, it is not totally dependable as the absence of rescue of a particular observed phenotype does not necessarily mean that the effect observed with the shRNA was due to off-target effects. Indeed, in ideal circumstances, every splice variant targeted by the shRNA should be restored in the rescue experiment, which might not always be realistic [24]. In this work, the reintroduction of the only known MPV17-coding transcript refuted any role of the protein in cancer cell proliferation.

In conclusion, our work demonstrates that MPV17 does not induce depletion of mtDNA content in cancer cell lines and that MPV17 does not control their cell cycle/proliferation. Importantly, in the future, rescue experiments should be a requirement in any study involving shRNA in order to silence a gene and analyse its subsequent effects on a particular phenotype. While the absence of rescue of the phenotype is not a strict indicator of aspecficity of the results, the restoration of the phenotype surely is a strong argument in favour of the specificity of any shRNA-induced phenotype of interest. Noteworhty, we believe that sh129921 requires further investigation as its robust effect on cell proliferation of different cancer cell lines could bring to light a promising actor that could be exploited in the fight against cancer.

## Materials and methods

### Cell culture

Cells were grown in Dulbecco′s Modified Eagle′s Medium (DMEM) low glucose (Life technologies, #31885) for human hepatoma cell lines Huh7 and HepG2 or in DMEM high glucose (Life technologies, #41965) for human renal embryonic cell line HEK293T, or in MEM GlutaMAX-1 (Life technologies, #42360) for human pulmonary adenocarcinoma cell line A549 and human squamous cell carcinoma cell line SQD9, or in Roswell Park Memorial Institute medium 1640 (Life technologies, #21875) for human hepatoma cell line Hep3B, supplemented with 10% foetal bovine serum (Life technologies, #10270) in a 5% CO_2_ humid atmosphere at 37°C. SQD9 cells were obtained from UCL (Vanessa Bol, Woluwe, Belgium). Huh7 cells (JCRB0403) were kindly provided by Prof. Sven Diederichs (DKFZ, Heidelberg, Germany). HepG2 (ATCC, HB-8065) were kindly provided by Prof. Luc Bertrand (UCL, Woluwe, Belgium). Hep3B were purchased from ATCC (HB-8064). A549 cells (ATCC, CCL-185) were kindly provided by Dr Jacques Piette (ULg, Liege, Belgium). HEK293T were obtained from ATCC (CRL-11268).

### Lentivirus production

HEK293T were seeded at 4x10^6^ cells in 75 cm^2^ culture flask (Corning, #430641U). The next day, the DNA mixture and the lipofectamine solution were prepared separately. The DNA mixture was composed of 0.4 μg of the envelope encoding vector pCMV-VSVG (Addgene, #8454), 3.6 μg of the packaging vector psPAX2 (Addgene, #12260) and 4 μg of the expressing plasmid (Table 1), in 240 μL of 5% opti-MEM (Invitrogen, #31985). The lipofectamine solution was composed of 16 μL of lipofectamine 2000 (Invitrogen, #11669) in 240 μL of 5% opti-MEM. Both preparations were incubated for 5 min at room temperature, combined, incubated for 30 min at room temperature and added in the flask. After 18 h, the medium was renewed and at 48 h and 72 h post-transfection, the medium was collected and filtered on 0.45 μm steriflip (Millipore, SE1M003M00). Lentiviruses were titrated by Reverse Transcription quantitative Polymerase Chain Reaction (RT-qPCR) according to manufacturer′s recommendations (Lentivirus qPCR Titer Kit, Applied Biological Materials, LV900).

**Table 1:** List of plasmids used for MPV17 silencing.

Vector	Reference
pLKO.1-puro sh131201	Sigma-Aldrich
	SHCLND-NM_002437
	TRCN0000131201
pLKO.1-puro sh128669	Sigma-Aldrich
	SHCLND-NM_002437
	TRCN0000128669
pLKO.1-puro sh127649	Sigma-Aldrich
	SHCLND-NM_002437
	TRCN0000127649
pLKO.1-puro sh129921	Sigma -Aldrich
	SHCLND-NM_002437
	TRCN0000129921
pLKO.1-puro sh131038	Sigma-Aldrich
	SHCLND-NM_002437
	TRCN0000131038
pLKO.1-puro shNT	Sigma-Aldrich
	SHC016-1EA
pLKO-puro-IPTG-3xLacO sh129921	Sigma-Aldrich
	09301606MN
	TRCN0000129921

The vectors backbone and encoding shRNA are specified along with their references.

### *MPV17* silencing

Sub-confluent cells were transduced in presence of 60 μg/mL protamine sulphate (Sigma-Aldrich, P4020). The next day, they were selected for 6 days with puromycin (Sigma-Aldrich, P8833) at 2.5 μg/mL. Experimental timelines of *MPV17* silencing with the constitutive and inducible vectors are detailed in S1 Fig. Briefly, for the constitutive silencing of *MPV17*, cells were transduced with different shRNA-encoding vectors (pLKO.1-puro shNT, sh127649, sh128669, sh129921, sh131201, sh131038), selected as described above and let to recover for 2 days without puromycin. They were then seeded and allowed to grow for 4 days before assessing the proliferation.

For the inducible silencing of *MPV17*, cells were transduced or not with pLKO-puro-IPTG-3xLacO encoding sh129921, selected as described above and let to recover for 2 days. *MPV17* silencing was then obtained by incubation of the transduced cells in the presence of 0.1 mM of IsoPropyl ß-D-1-ThioGalactopyranoside (IPTG) (Sigma-Aldrich, I6758), renewed daily for 5 days unless stated otherwise. Cells were then seeded and allowed to grow for 4 days in presence of IPTG renewed daily (unless stated otherwise) before assessing the proliferation.

### Proliferation assessment

#### Cell counting

Cells were seeded in 25 cm^2^ culture flask (Corning, #430639) and grown for 4 days. Cells were rinsed with Phosphate Buffered Saline (PBS) pH 7.4, trypsinized with 0.05% trypsin-EDTA (ThermoFisher, 25300) and centrifuged for 5 min at 200 g. The pellet was resuspended in culture medium and cell suspension density was counted in a Neubauer chamber. Doubling time was calculated as follow: time*ln(2) / (ln(number of cells at the end of the experiment)—ln(number of seeded cells)).

#### MTT assay

Cells were seeded in 24-well culture plates (Corning, #3524), grown for 4 days and incubated for 1 h with 500 μL of tetrazolium dye MTT (Sigma-Aldrich, M2128) (2.5 μg/mL in PBS) at 37°C. Cells were then lysed for 1 h in lysis buffer (9% sodium dodecyl sulphate, 60% N, N-dimethylformamide, pH 4.7), and absorbance was measured with a spectrophotometer (xMark, Bio-Rad) at 570 nm.

#### Protein content: Folin protein assay

Cells were seeded in 24-well culture plates (Corning, #3524), grown for 4 days and rinsed twice with PBS. The bovine serum albumin (VWR, 0332) protein standards and samples were incubated 30 min in presence of 200 μL of 0.5 M sodium hydroxide, then 10 min with 750 μL of a solution A (1.96% sodium carbonate, 0.02% sodium and potassium tartrate and 0.01% copper sulphate) and finally 30 min with 75 μL of Folin and Ciocalteu′s phenol reagent (Sigma-Aldrich, F9252) diluted twice in distilled water. Absorbance was measured with a spectrophotometer (xMark, Bio-Rad) at 740 nm [25].

### Cell lysates and pierce protein assay

Cells were seeded in 75 cm^2^ culture flask (Corning, #430641U), rinsed once with PBS and lysed with radioimmunoprecipitation assay buffer (20 mM tris hydroxymethyl, 150 mM sodium chloride, 1 mM EDTA, 1 mM EGTA, 1% sodium deoxycholate, 10% glycerol, 1% NP40, pH 7.6) supplemented with protease inhibitor cocktail (Sigma-Aldrich, 11697498001) and phosphatase inhibitor buffer (25 mM sodium orthovanadate, 250 mM 4-nitrophenylphosphate, 250 mM ß-glycerophosphate, 125 mM sodium fluoride). Lysates were sonicated 3 x 10 sec (amplitude 50) and centrifuged (10 min, 15000 g). Cleared cell lysates were assessed for protein content with Pierce 660 Protein Assay Reagent (ThermoFisher, 22660) according to the manufacturer′s recommendations.

### Western blotting analysis

Amounts of 20 μg of protein samples were prepared in loading buffer (0.03 M Tris-hydrochloride acid; pH 6.8, 0.04 M sodium dodecyl sulphate, 0.4 M β-mercaptoethanol, 5% glycerol, 0.15 mM bromophenol blue), boiled for 5 min, resolved on polyacrylamide gel and transferred on a nitrocellulose membrane (Bio-Rad). Membrane was blocked for 1 h at room temperature in Odyssey Blocking Buffer (OBB) (LI-COR, P/N 927) diluted twice in PBS and incubated overnight at 4°C with the primary antibody diluted in OBB with 0.1% Tween-20 (OBB-T). For MPV17 detection, membrane was treated prior to blocking step with Super Signal Western Blot Enhancer (ThermoFisher, 46640) according to the manufacturer′s recommendations. The next day, membrane was rinsed 3 x 5 min in PBS with 0.1% Tween-20 (PBS-T), incubated with secondary antibody diluted in OBB-T 0.1% for 1 h at room temperature, rinsed 3 times in PBS-T 0.1%, dried and scanned with the Odyssey Infrared Imager (LI-COR, 9120). For the description of the antibodies used in this study, see Table 2.

**Table 2:** List of antibodies and their dilutions used for western blotting.

Antibody	Reference	Dilution
Anti-MPV17	Proteintech	1/1000
	10310-1-AP	
Anti-ATF4	Santa Cruz	1/1000
	Sc-390063	
Anti-ATF4	Cell Signaling	1/1000
	#11815 lot 3	
Anti-tubulin	Sigma-Aldrich	1/10000
	T5168	
Anti -actin	Sigma-Aldrich	1/10000
	A5441	
Anti-GAPDH	Abcam	1/10000
	128915	
Anti-rabbit IgG	LI-COR	1/10000
IRDye 800CW Goat	926–32211	
Anti-mouse IgG	LI-COR	1/10000
IRDye 680RD Goat	926–68070	
Anti-rabbit IgG	LI-COR	1/10000
IRDye 680RD Goat	926–68071	
Anti-mouse IgG	LI-COR	1/10000
IRDye 800CW Goat	926–32210	

The antibodies used for the western blot analysis are specified with their reference and working dilutions.

### Construct for MPV17 overexpression

MPV17 mRNA was reverse transcribed (Transcriptor First Strand cDNA kit, Roche, 04379012001) using a specific primer (5′-AGGTGGAAACGATGGAGTGA-3′). A PCR was then performed using a forward primer containing a restriction site for BamH1 (F: 5′-AGGATCCAGGAAGCATGGCA-3′) and a reverse primer containing a restriction site for Sal1 (R: 5′-AGTCGACGGCAGGCTTAGA-3′). PCR products were purified using Wizard SV Gel and PCR Clean-Up System (Promega, A9281). An amount of 1 μg of PCR product and pLenti PGK GFP Puro (Addgene, #19070) was digested with BamH1, purified, restricted with Sal1, purified and finally ligated with T DNA ligase (Biolabs, M0202S) to construct the pLenti PGK MPV17 Puro plasmid.

### Rescue experiment

HepG2 cells were transduced with pLenti PGK GFP puro or pLenti PGK MPV17 puro-containing lentiviruses and selected for 6 days with puromycin (2.5 μg/mL). Cells were then transduced with PLKO.1-puro vector constitutively encoding sh129921 or shNT (Table 1). Cells were then allowed to recover and generate *MPV17*-targeting shRNA for 5 days, seeded and allowed to grow for 4 days. Cell proliferation was assessed by MTT assay at day 1 and day 4. To overexpress MPV17, we used *MPV17*-silencing and *MPV17*-overexpression vectors that were both bearing the resistance to puromycin. This obviously constitutes an obstacle in the selection of the cells that are double transduced. However, we decided to proceed further based on the knowledge that the sh129921-encoding vector robustly led to a very efficient transduction rate (nearly 100%), therefore allowing to bypass the need for an ensuing antibiotic selection.

### mtDNA content determination

DNA was extracted with the Wizard Genomic DNA Purification Kit (Promega, A1120) according to the manufacturer′s recommendations. qPCR for mtDNA amplification was performed on the gene encoding ND2 (NADH Dehydrogenase 2) using the forward primer 5′-TGTTGGTTATACCCTTCCCGTACTA-3′ and the reverse primer 5′-CCTGCAAAGATGGTAGAGTAGATGA-3′. For the normalization with nuclear DNA, the gene encoding Beclin was amplified with the forward primer 5′-CCCTCATCACAGGGCTCTCTCCA-3′ and the reverse primer 5′- GGGACTGTAGGCTGGGAACTATGC-3′. Real time PCR was performed with SYBR Select Master Mix (ThermoFisher, 4472908). mtDNA copy number was calculated according to the following formula: 2*2^^-ΔCt^ (where ΔCt = Ctmean _ND2_ –Ctmean _Beclin_).

### RNA extraction and RT-qPCR

RNA was extracted with RNeasy Mini kit (Qiagen, 74104) according to manufacturer′s recommendations and QIAcube (Qiagen). RT was performed with GoScript™ Reverse Transcription Mix (Promega, A2791) according to the manufacturer′s recommendations. qPCR was performed with SYBR Select Master Mix (ThermoFisher, 4472908). We used the 2^-ΔΔCt^ method to assess the relative mRNA expression. The *MPV17* primers used in this study are the following: F: GCTCAGGAAGCATGGCACTCT; R: AATGTCACCCAGGCCCATCA.

### RNA sequencing

Huh7 cells were transduced with shRNA non-target lentiviral vector (shNT) or with shRNA vector constitutively targeting *MPV17* expression (sh129921). RNA quality was analysed with the Bioanalyzer 2100 (Agilent). RNA samples (*n* = 4) were sent to Genomic Core Leuven (Belgium) for RNA sequencing and data were analysed with Ingenuity Pathway Analysis (QIAGEN Inc., https://www.qiagenbioinformatics.com/products/ingenuitypathway-analysis).

### Immunohistochemistry

Slices were incubated 2 x 5 min in xylene (ThermoFisher, X/0200/21), 2 x 3 min in isopropanol (VWR, 20842.330) and 10 min in 1% H_2_O_2_ (VWR, 23.613.446) / methanol prepared extemporaneously. Slices were washed 3 min in tap water and 3 min in demineralized water. Slices were then incubated 30 min in the 98°C water bath with the “Target Retrieval Solution 1X” pH 6.1 (Dako, S169984-2) and let to cool down for 15 min at room temperature. Slices were washed 5 min in tap water, 2 x 3 min in PBS and incubated 1 h at room temperature with a solution of PBS-2% Normal Goat Serum (NGS) (ThermoFisher, 16210064). Slices were then incubated with the primary antibody anti-MPV17 (Table 2) diluted 1/100 in a solution of PBS-0.5% NGS, overnight at 4°C. Slices were washed 3 x 3 min in PBS-T 0.05% baths and 3 min in PBS. Slices were then incubated 30 min at room temperature with the secondary antibody EnVision-HRP anti-rabbit (Dako, K400311). Slices were washed 3 x 3 min in PBS-T 0.05% and 3 min in PBS. Slices were then incubated 4 min at room temperature in the DAB solution (Dako, K346811) and washed 5 min in running tap water. Slices were incubated 5 min in Mayer Hematoxyline and washed 5 min in running tap water. Slices were finally incubated 3x 3 min in isopropanol, 3x 3 min in xylene and mounted on coverslip with Entallan glue.

### Statistical analysis

Data are expressed as the mean ± Standard Error of the Mean (S.E.M). Figure plotting was performed with Prism software. Statistical analysis was performed using a one-tailed Mann-Whitney Test or a two-tailed Wilcoxon signed rank Test (⍺ = 5%; *: p<0.05; **: p<0.01; ***: p<0.001; ****: p<0.0001).

## Supporting information

S1 Fig Experimental timelines of *MPV17* silencing in the constitutive and inducible models. Cells were transduced and puromycin-selected with a vector allowing a constitutive (a) or an inducible (b) *MPV17* silencing. After 2 days of recovery, cells were either seeded and allowed to grow for 4 days before assessment of proliferation (a) or treated with 0.1 mM of IsoPropyl ß-D-1-ThioGalactopyranoside (IPTG) for 5 days to induce *MPV17* silencing prior to the seeding (b). The proliferation was then assessed after 4 days of growth in presence of IPTG renewed daily.
(PPTX)

S2 Fig Effect of IPTG on Huh7 cells proliferation following constitutive sh129921-mediated *MPV17* knockdown. Huh7 cells were transduced with non-target shRNA lentiviral vectors (shNT) or with sh129921-containing vector. Transduced cells were selected for 6 days with puromycin (2.5 μg/mL). Cells were seeded at 8×10^3^ cells/cm^2^ and grown for 4 days in the presence or in the absence of 0.1 mM of IPTG. To mimic the conditions found in the inducible model of expression, the IPTG-containing medium was renewed daily. We therefore included a control in which no IPTG was present while the medium was also changed every day (Ctl). Data are presented as mean ± S.E.M (3 biological replicates). P values were calculated with the one-tailed Mann-Whitney Test (⍺ = 5%; NS; *: p<0.05; **: p<0.01; ***: p<0.001).
(PPTX)

S3 Fig Effect of the duration of IPTG pre-treatment and of the medium change frequency on Huh7 cells proliferation in response to inducible sh129921-mediated *MPV17* knockdown. Cells were transduced, or not (Unt), with inducible sh129921 lentiviral vectors and selected for 6 days with puromycin (2.5 μg/mL). Cells were then incubated for 14 days in the presence of 0.1 mM of IPTG to induce *MPV17* silencing and culture medium was changed daily (a, b, c) or every 2 days (c, d, e). Cells were seeded at 8×10^3^ cells/cm^2^ and grown for 4 days in presence of IPTG in the same conditions. MPV17 protein abundance was assessed by western blot analysis (a, d) and quantified with Image J software (b, e). Proliferation was then assessed by manual counting to calculate the doubling time (c, f). Full blots are presented in S6 Fig. Data are presented as mean ± S.E.M of 3 independent biological replicates. P values were calculated with the one-tailed Mann-Whitney Test (⍺ = 5%; NS; *: p<0.05; **: p<0.01; ***: p<0.001).
(PPTX)

S4 Fig Localisation of the shRNA-targeted sites on *MPV17* transcript. The effects of several commercially available shRNAs directed against *MPV17* transcript were assessed in Huh7 cells. Each shRNA Sigma Aldrich reference (sh128669, sh131201, sh131038, sh127649 and sh129921) is indicated above its target site. Each grey box represents an exon of *MPV17* transcript (NM002437.5). A line indicates the 3′UTR and 5′UTR of *MPV17* transcript.
(PPTX)

S5 Fig Localisation of the shRNA-targeted sites on the *MPV17* transcript isoforms referenced in human liver. The Genotype-Tissue Expression (GTEx) Project was supported by the Common Fund of the Office of the Director of the National Institutes of Health, and by NCI, NHGRI, NHLBI, NIDA, NIMH, and NINDS. The data used for the analysis described in this manuscript and this Fig. were obtained from the GTEx Portal on 02/05/19. We indicated the Sigma Aldrich reference of each shRNA targeting *MPV17* transcripts (sh128669, sh131201, sh131038, sh127649 and sh129921) above its targeted site. Each box represents an exon in *MPV17* transcript isoforms. The darker the purple, the more abundant the transcript (as referred by Log 10 (TPM)). The 3′UTR is located on the left of the image, and the 5′UTR on the right. We added an asterisk (*) that indicates both shRNAs providing the reduced proliferation phenotype, while ø indicates both shRNAs leading to an unchanged proliferation rate.
(PPTX)

S6 Fig Full-length blots of the cropped blots presented in this work. Protein immunostaining was performed with secondary antibodies coupled to infrared dyes (IRDye; green: 800nm/red: 700 nm). a: Full blot of the one presented in Figs 2A and 7A; b: Full blot of the one presented in Figs 4A and 7B (Anti-ATF4 from Santa Cruz); c: Full blot of the one presented in Fig 8A; d: Full blot of the one presented in Fig 4C (Anti-ATF4 from Cell Signaling); e: Full blot of the one presented in Fig 4B (Anti-ATF4 from Cell Signaling); f: Full blot of the one presented in Fig 5B; g: Full blot of the one presented in Fig 6B; h and i: Full blots of the ones presented in S3 Fig; j: Full blot of the one presented in Fig 6C (Anti-ATF4 from Santa Cruz).
(DOCX)

## References

[1] Karasawa M, Zwacka RM, Reuter A, Fink T, Hsieh CL, Lichter P, et al The human homolog of the glomerulosclerosis gene Mpv17: structure and genomic organization. Hum Mol Genet. Oxford University Press; 1993;2: 1829–1834. 10.1093/hmg/2.11.1829 8281143

[2] Spinazzola A, Viscomi C, Fernandez-Vizarra E, Carrara F, D′Adamo P, Calvo S, et al MPV17 encodes an inner mitochondrial membrane protein and is mutated in infantile hepatic mitochondrial DNA depletion. Nat Genet. Nature Publishing Group; 2006;38: 570–575. 10.1038/ng1765 16582910

[3] Krauss J, Astrinidis P, Astrinides P, Frohnhöfer HG, Walderich B, Nüsslein-Volhard C. transparent, a gene affecting stripe formation in Zebrafish, encodes the mitochondrial protein Mpv17 that is required for iridophore survival. Biol Open. Company of Biologists; 2013;2: 703–10. 10.1242/bio.20135132 23862018 PMC3711038

[4] Trott A, Morano KA. SYM1 Is the Stress-Induced Saccharomyces cerevisiae Ortholog of the Mammalian Kidney Disease Gene Mpv17 and Is Required for Ethanol Metabolism and Tolerance during Heat Shock. Eukaryot Cell. American Society for Microbiology (ASM); 2004;3: 620 10.1128/EC.3.3.620-631.2004 15189984 PMC420134

[5] El-Hattab AW, Wang J, Dai H, Almannai M, Staufner C, Alfadhel M, et al *MPV17*-related mitochondrial DNA maintenance defect: New cases and review of clinical, biochemical, and molecular aspects. Hum Mutat. 2018;39: 461–470. 10.1002/humu.23387 29282788

[6] Löllgen S, Weiher H. The role of the Mpv17 protein mutations of which cause mitochondrial DNA depletion syndrome (MDDS): lessons from homologs in different species. Biol Chem. De Gruyter; 2015;396: 13–25. 10.1515/hsz-2014-0198 25205723

[7] Antonenkov VD, Isomursu A, Mennerich D, Vapola MH, Weiher H, Kietzmann T, et al The Human Mitochondrial DNA Depletion Syndrome Gene MPV17 Encodes a Non-selective Channel That Modulates Membrane Potential. J Biol Chem. American Society for Biochemistry and Molecular Biology; 2015;290: 13840–61. 10.1074/jbc.M114.608083 25861990 PMC4447960

[8] Reinhold R, Kruger V, Meinecke M, Schulz C, Schmidt B, Grunau SD, et al The Channel-Forming Sym1 Protein Is Transported by the TIM23 Complex in a Presequence-Independent Manner. Mol Cell Biol. 2012;32: 5009–5021. 10.1128/MCB.00843-12 23045398 PMC3510547

[9] Dalla Rosa I, Cámara Y, Durigon R, Moss CF, Vidoni S, Akman G, et al MPV17 Loss Causes Deoxynucleotide Insufficiency and Slow DNA Replication in Mitochondria. Larsson N-G, editor. PLOS Genet. Public Library of Science; 2016;12: e1005779 10.1371/journal.pgen.1005779 26760297 PMC4711891

[10] Moss CF, Dalla Rosa I, Hunt LE, Yasukawa T, Young R, Jones AWE, et al Aberrant ribonucleotide incorporation and multiple deletions in mitochondrial DNA of the murine MPV17 disease model. Nucleic Acids Res. 2017;45: 12808–12815. 10.1093/nar/gkx1009 29106596 PMC5728394

[11] Martorano L, Peron M, Laquatra C, Lidron E, Facchinello N, Meneghetti G, et al The zebrafish orthologue of the human hepatocerebral disease gene MPV17 plays pleiotropic roles in mitochondria. Dis Model Mech. Company of Biologists; 2019;12 10.1242/dmm.037226 30833296 PMC6451431

[12] Alonzo JR, Venkataraman C, Field MS, Stover PJ. The mitochondrial inner membrane protein MPV17 prevents uracil accumulation in mitochondrial DNA. J Biol Chem. 2018;293: 20285–20294. 10.1074/jbc.RA118.004788 30385507 PMC6311524

[13] Wanet A, Caruso M, Domelevo Entfellner J-B, Najar M, Fattaccioli A, Demazy C, et al The Transcription Factor 7-Like 2-Peroxisome Proliferator-Activated Receptor Gamma Coactivator-1 Alpha Axis Connects Mitochondrial Biogenesis and Metabolic Shift with Stem Cell Commitment to Hepatic Differentiation. Stem Cells. 2017;35: 2184–2197. 10.1002/stem.2688 28795454

[14] Choi Y-R, Hong Y Bin, Jung S-C, Lee JH, Kim YJ, Park HJ, et al A novel homozygous MPV17 mutation in two families with axonal sensorimotor polyneuropathy. BMC Neurol. BioMed Central; 2015;15: 179 10.1186/s12883-015-0430-1 26437932 PMC4595119

[15] Dallabona C, Marsano RM, Arzuffi P, Ghezzi D, Mancini P, Zeviani M, et al Sym1, the yeast ortholog of the MPV17 human disease protein, is a stress-induced bioenergetic and morphogenetic mitochondrial modulator. Hum Mol Genet. Narnia; 2010;19: 1098–1107. 10.1093/hmg/ddp581 20042463

[16] Bottani E, Giordano C, Civiletto G, Di Meo I, Auricchio A, Ciusani E, et al AAV-mediated liver-specific MPV17 expression restores mtDNA levels and prevents diet-induced liver failure. Mol Ther. American Society of Gene & Cell Therapy; 2014;22: 10–7. 10.1038/mt.2013.230 24247928 PMC3880585

[17] Wortel IMN, van der Meer LT, Kilberg MS, van Leeuwen FN. Surviving Stress: Modulation of ATF4-Mediated Stress Responses in Normal and Malignant Cells. Trends Endocrinol Metab. 2017;28: 794–806. 10.1016/j.tem.2017.07.003 28797581 PMC5951684

[18] Harding HP, Zhang Y, Zeng H, Novoa I, Lu PD, Calfon M, et al An Integrated Stress Response Regulates Amino Acid Metabolism and Resistance to Oxidative Stress. Mol Cell. Cell Press; 2003;11: 619–633. 10.1016/s1097-2765(03)00105-9 12667446

[19] Malmberg SE, Adams CM. Insulin signaling and the general amino acid control response. Two distinct pathways to amino acid synthesis and uptake. J Biol Chem. American Society for Biochemistry and Molecular Biology; 2008;283: 19229–34. 10.1074/jbc.M801331200 18480057

[20] Wang S, Chen XA, Hu J, Jiang J-K, Li Y, Chan-Salis KY, et al ATF4 Gene Network Mediates Cellular Response to the Anticancer PAD Inhibitor YW3-56 in Triple-Negative Breast Cancer Cells. Mol Cancer Ther. NIH Public Access; 2015;14: 877–88. 10.1158/1535-7163.MCT-14-1093-T 25612620 PMC4394025

[21] Ben-Sahra I, Hoxhaj G, Ricoult SJH, Asara JM, Manning BD. mTORC1 induces purine synthesis through control of the mitochondrial tetrahydrofolate cycle. Science. NIH Public Access; 2016;351: 728–733. 10.1126/science.aad0489 26912861 PMC4786372

[22] Politi N, Pasotti L, Zucca S, Casanova M, Micoli G, Cusella De Angelis M, et al Half-life measurements of chemical inducers for recombinant gene expression. J Biol Eng. 2014;8: 5 10.1186/1754-1611-8-5 24485151 PMC3940292

[23] RNA Interference and Viruses: Current Innovations and Future Trends—Google Books [Internet]. [cited 27 Feb 2019]. Available: https://books.google.be/books?id=C5TY8W74scIC&pg=PA204&lpg=PA204&dq=inducible+vector+reversible+fine+tuning&source=bl&ots=j8lvhrcKPL&sig=ACfU3U0knMfwhxsLArPPpV4QXORxuiAuHQ&hl=en&sa=X&ved=2ahUKEwjakOO159vgAhWTQhUIHa0OA4IQ6AEwEHoECAAQAQ#v=onepage&q=inducibl

[24] Peretz L, Besser E, Hajbi R, Casden N, Ziv D, Kronenberg N, et al Combined shRNA over CRISPR/cas9 as a methodology to detect off-target effects and a potential compensatory mechanism. Sci Rep. Nature Publishing Group; 2018;8: 93 10.1038/s41598-017-18551-z 29311693 PMC5758708

[25] LOWRY OH, ROSEBROUGH NJ, FARR AL, RANDALL RJ. Protein measurement with the Folin phenol reagent. J Biol Chem. 1951;193: 265–75. Available: http://www.ncbi.nlm.nih.gov/pubmed/14907713 14907713

