# Supplementary material for: MPV17 does not control cancer cell proliferation
Source: PLoS One. 2020 Mar 10;15(3):e0229834. doi: 10.1371/journal.pone.0229834 (PMC7064194; doi:10.1371/journal.pone.0229834)
Supplement: S1 Fig — Cells were transduced and puromycin-selected with a vector allowing a constitutive (a) or an inducible (b) MPV17 silencing. After 2 days of recovery, cells were either seeded and allowed to grow for 4 days before assessment of proliferation (a) or treated with 0.1 mM of IsoPropyl ß-D-1-ThioGalactopyranoside (IPTG) for 5 days to induce MPV17 silencing prior to the seeding (b). The proliferation was then assessed after 4 days of growth in presence of IPTG renewed daily. (PPTX) [file pone.0229834.s001.pptx]

## Slide 1
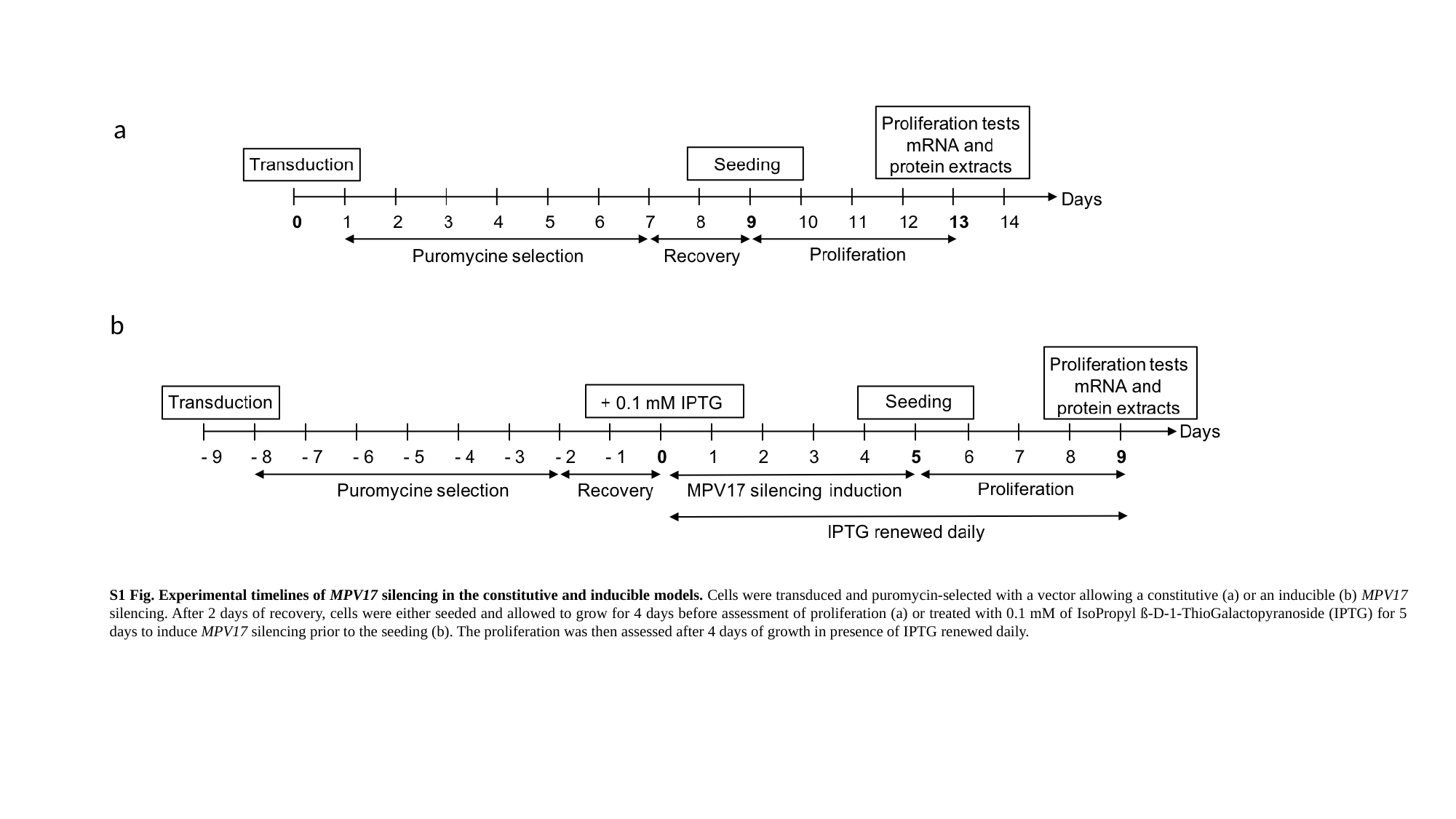

a
b
S1 Fig. Experimental timelines of MPV17 silencing in the constitutive and inducible models. Cells were transduced and puromycin-selected with a vector allowing a constitutive (a) or an inducible (b) MPV17 silencing. After 2 days of recovery, cells were either seeded and allowed to grow for 4 days before assessment of proliferation (a) or treated with 0.1 mM of IsoPropyl ß-D-1-ThioGalactopyranoside (IPTG) for 5 days to induce MPV17 silencing prior to the seeding (b). The proliferation was then assessed after 4 days of growth in presence of IPTG renewed daily.
