# Supplementary material for: MPV17 does not control cancer cell proliferation
Source: PLoS One. 2020 Mar 10;15(3):e0229834. doi: 10.1371/journal.pone.0229834 (PMC7064194; doi:10.1371/journal.pone.0229834)
Supplement: S2 Fig — Huh7 cells were transduced with non-target shRNA lentiviral vectors (shNT) or with sh129921-containing vector. Transduced cells were selected for 6 days with puromycin (2.5 μg/mL). Cells were seeded at 8×10^3^ cells/cm2 and grown for 4 days in the presence or in the absence of 0.1 mM of IPTG. To mimic the conditions found in the inducible model of expression, the IPTG-containing medium was renewed daily. We therefore included a control in which no IPTG was present while the medium was also changed every day (Ctl). Data are presented as mean ± S.E.M (3 biological replicates). P values were calculated with the one-tailed Mann-Whitney Test (⍺ = 5%; NS; *: p<0.05; **: p<0.01; ***: p<0.001). (PPTX) [file pone.0229834.s002.pptx]

## Slide 1
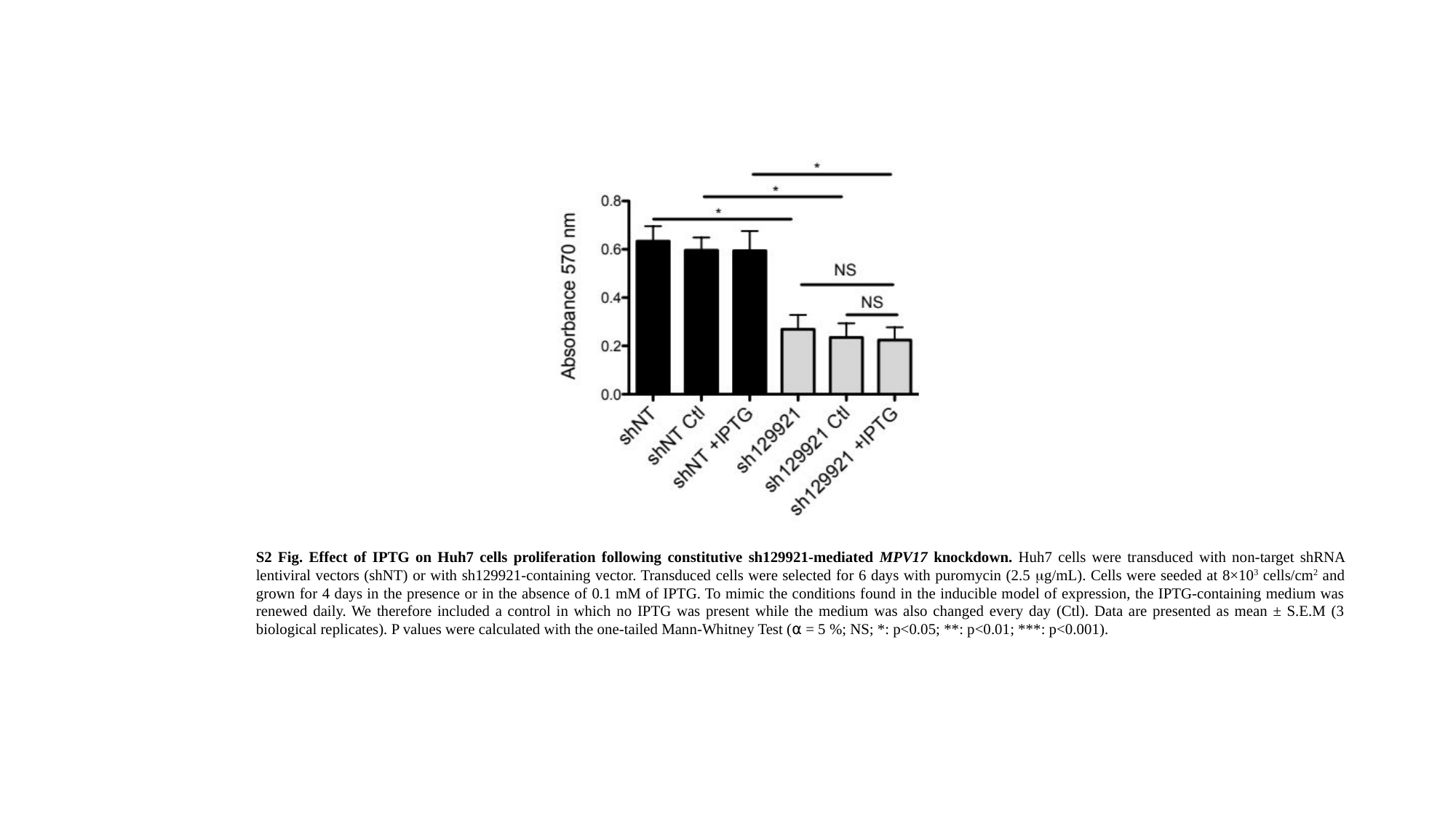

S2 Fig. Effect of IPTG on Huh7 cells proliferation following constitutive sh129921-mediated MPV17 knockdown. Huh7 cells were transduced with non-target shRNA lentiviral vectors (shNT) or with sh129921-containing vector. Transduced cells were selected for 6 days with puromycin (2.5 μg/mL). Cells were seeded at 8×10^3^ cells/cm2 and grown for 4 days in the presence or in the absence of 0.1 mM of IPTG. To mimic the conditions found in the inducible model of expression, the IPTG-containing medium was renewed daily. We therefore included a control in which no IPTG was present while the medium was also changed every day (Ctl). Data are presented as mean ± S.E.M (3 biological replicates). P values were calculated with the one-tailed Mann-Whitney Test (⍺ = 5 %; NS; *: p<0.05; **: p<0.01; ***: p<0.001).
