# Supplementary material for: MPV17 does not control cancer cell proliferation
Source: PLoS One. 2020 Mar 10;15(3):e0229834. doi: 10.1371/journal.pone.0229834 (PMC7064194; doi:10.1371/journal.pone.0229834)
Supplement: S3 Fig — Cells were transduced, or not (Unt), with inducible sh129921 lentiviral vectors and selected for 6 days with puromycin (2.5 μg/mL). Cells were then incubated for 14 days in the presence of 0.1 mM of IPTG to induce MPV17 silencing and culture medium was changed daily (a, b, c) or every 2 days (c, d, e). Cells were seeded at 8×10^3^ cells/cm2 and grown for 4 days in presence of IPTG in the same conditions. MPV17 protein abundance was assessed by western blot analysis (a, d) and quantified with Image J software (b, e). Proliferation was then assessed by manual counting to calculate the doubling time (c, f). Full blots are presented in S6 Fig. Data are presented as mean ± S.E.M of 3 independent biological replicates. P values were calculated with the one-tailed Mann-Whitney Test (⍺ = 5%; NS; *: p<0.05; **: p<0.01; ***: p<0.001). (PPTX) [file pone.0229834.s003.pptx]

## Slide 1
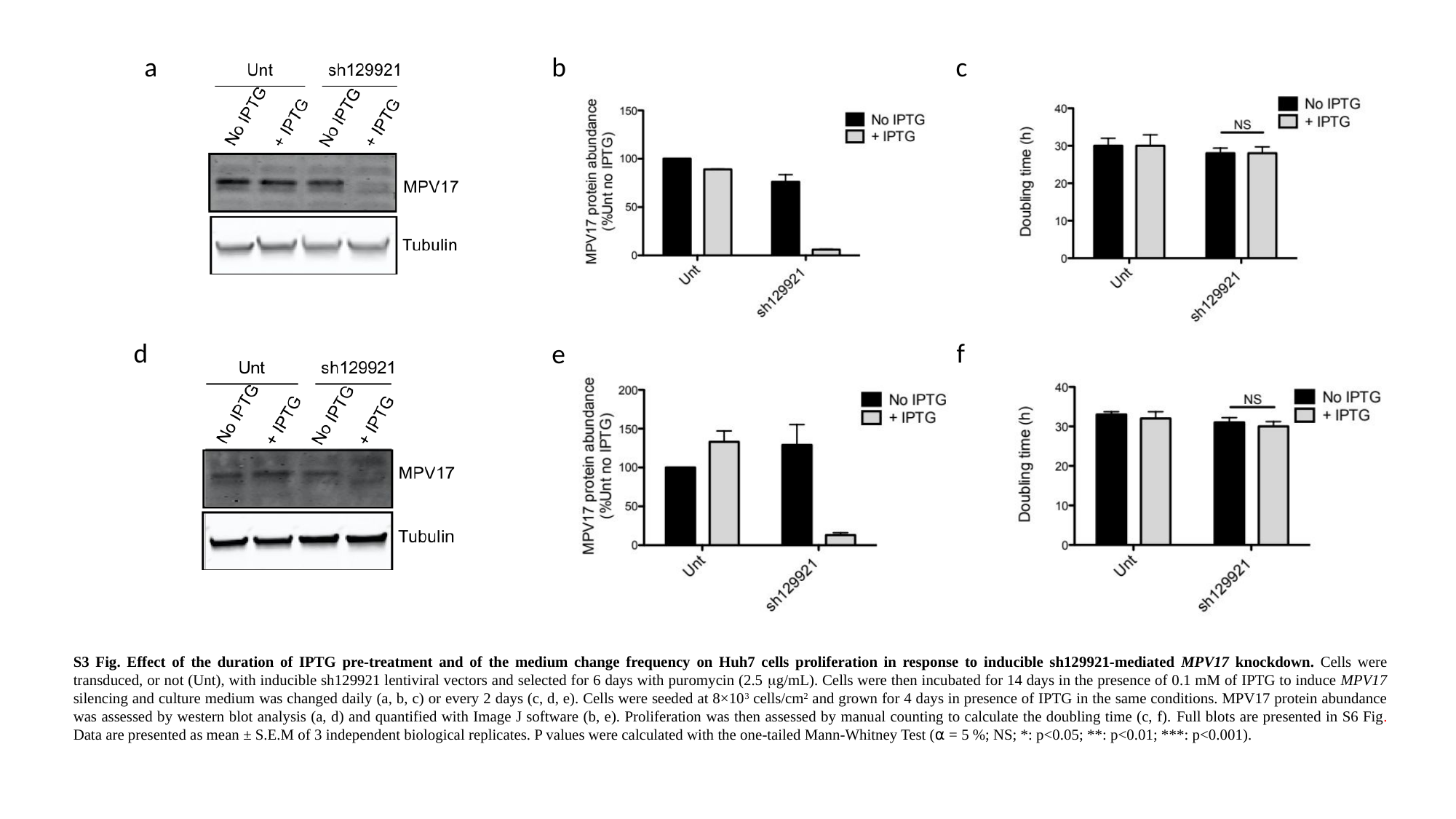

a
b
c
d
f
e
S3 Fig. Effect of the duration of IPTG pre-treatment and of the medium change frequency on Huh7 cells proliferation in response to inducible sh129921-mediated MPV17 knockdown. Cells were transduced, or not (Unt), with inducible sh129921 lentiviral vectors and selected for 6 days with puromycin (2.5 μg/mL). Cells were then incubated for 14 days in the presence of 0.1 mM of IPTG to induce MPV17 silencing and culture medium was changed daily (a, b, c) or every 2 days (c, d, e). Cells were seeded at 8×10^3^ cells/cm2 and grown for 4 days in presence of IPTG in the same conditions. MPV17 protein abundance was assessed by western blot analysis (a, d) and quantified with Image J software (b, e). Proliferation was then assessed by manual counting to calculate the doubling time (c, f). Full blots are presented in S6 Fig. Data are presented as mean ± S.E.M of 3 independent biological replicates. P values were calculated with the one-tailed Mann-Whitney Test (⍺ = 5 %; NS; *: p<0.05; **: p<0.01; ***: p<0.001).
