# Supplementary material for: MPV17 does not control cancer cell proliferation
Source: PLoS One. 2020 Mar 10;15(3):e0229834. doi: 10.1371/journal.pone.0229834 (PMC7064194; doi:10.1371/journal.pone.0229834)
Supplement: S4 Fig — The effects of several commercially available shRNAs directed against MPV17 transcript were assessed in Huh7 cells. Each shRNA Sigma Aldrich reference (sh128669, sh131201, sh131038, sh127649 and sh129921) is indicated above its target site. Each grey box represents an exon of MPV17 transcript (NM002437.5). A line indicates the 3′UTR and 5′UTR of MPV17 transcript. (PPTX) [file pone.0229834.s004.pptx]

## Slide 1
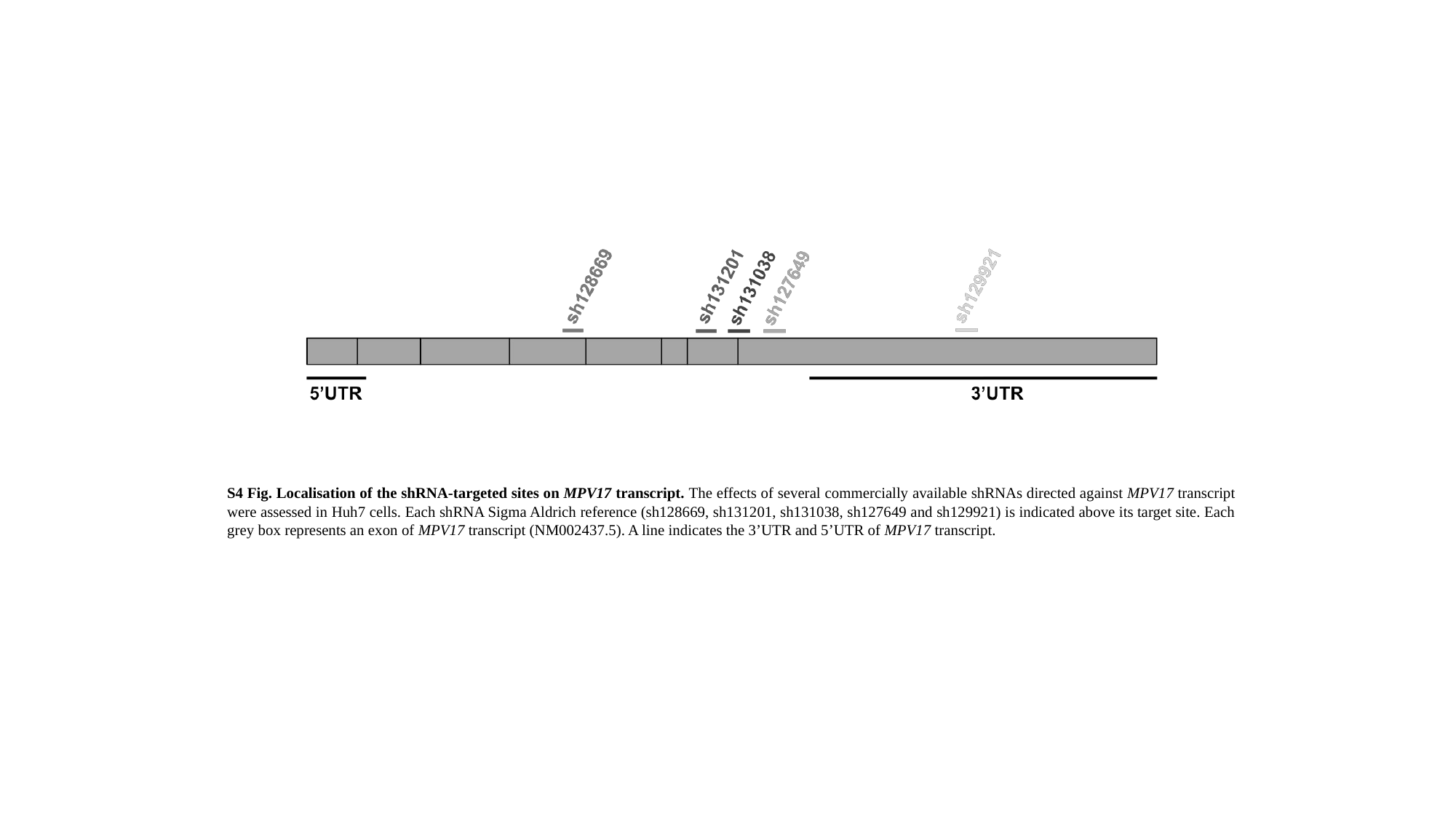

S4 Fig. Localisation of the shRNA-targeted sites on MPV17 transcript. The effects of several commercially available shRNAs directed against MPV17 transcript were assessed in Huh7 cells. Each shRNA Sigma Aldrich reference (sh128669, sh131201, sh131038, sh127649 and sh129921) is indicated above its target site. Each grey box represents an exon of MPV17 transcript (NM002437.5). A line indicates the 3’UTR and 5’UTR of MPV17 transcript.
