# Supplementary material for: MPV17 does not control cancer cell proliferation
Source: PLoS One. 2020 Mar 10;15(3):e0229834. doi: 10.1371/journal.pone.0229834 (PMC7064194; doi:10.1371/journal.pone.0229834)
Supplement: S5 Fig — The Genotype-Tissue Expression (GTEx) Project was supported by the Common Fund of the Office of the Director of the National Institutes of Health, and by NCI, NHGRI, NHLBI, NIDA, NIMH, and NINDS. The data used for the analysis described in this manuscript and this Fig. were obtained from the GTEx Portal on 02/05/19. We indicated the Sigma Aldrich reference of each shRNA targeting MPV17 transcripts (sh128669, sh131201, sh131038, sh127649 and sh129921) above its targeted site. Each box represents an exon in MPV17 transcript isoforms. The darker the purple, the more abundant the transcript (as referred by Log 10 (TPM)). The 3′UTR is located on the left of the image, and the 5′UTR on the right. We added an asterisk (*) that indicates both shRNAs providing the reduced proliferation phenotype, while ø indicates both shRNAs leading to an unchanged proliferation rate. (PPTX) [file pone.0229834.s005.pptx]

## Slide 1
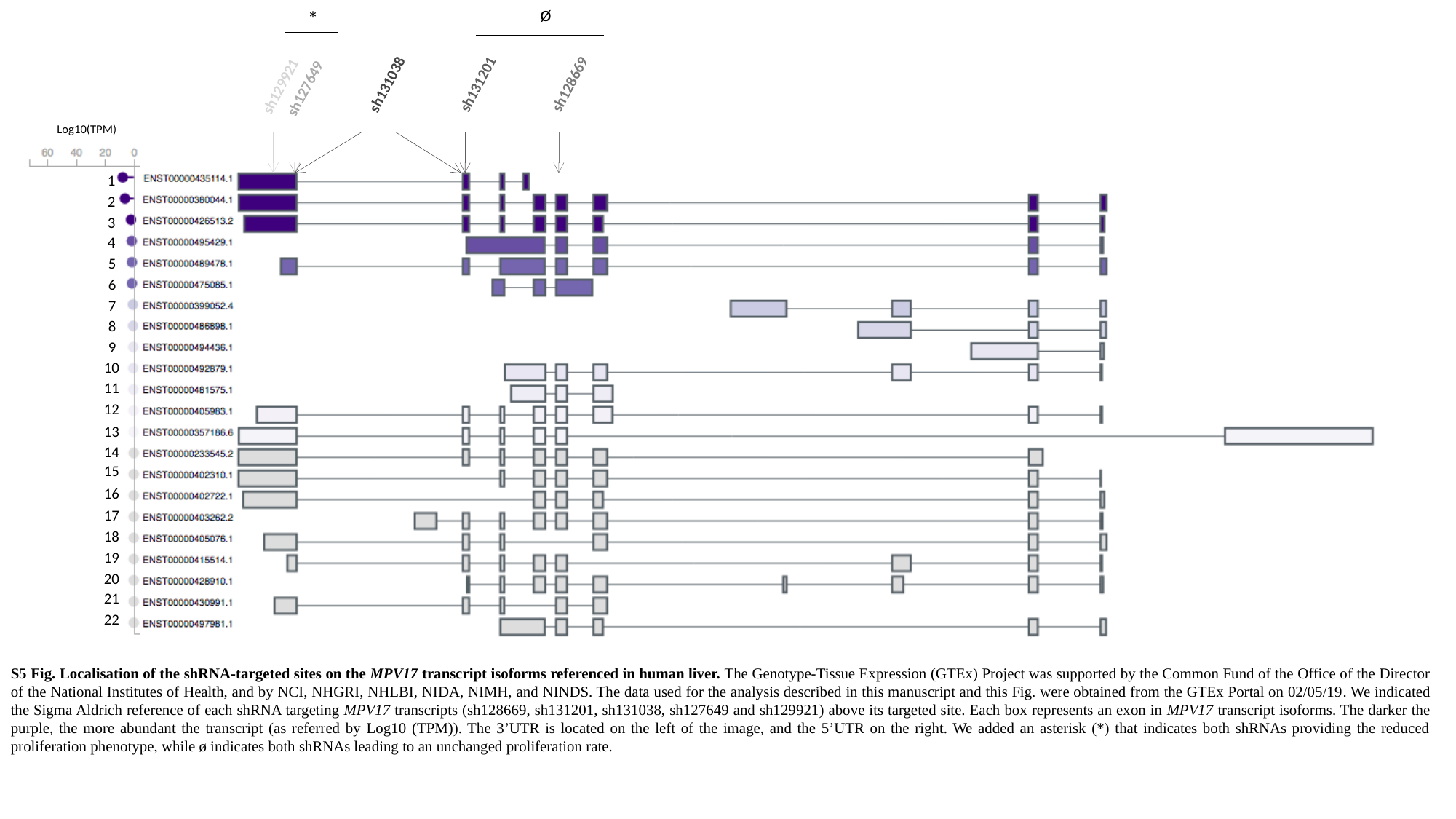

ø
*
sh131201
sh128669
sh131038
sh129921
sh127649
Log10(TPM)
1
2
3
4
5
6
7
8
9
10
11
12
13
14
15
16
17
18
19
20
21
22
S5 Fig. Localisation of the shRNA-targeted sites on the MPV17 transcript isoforms referenced in human liver. The Genotype-Tissue Expression (GTEx) Project was supported by the Common Fund of the Office of the Director of the National Institutes of Health, and by NCI, NHGRI, NHLBI, NIDA, NIMH, and NINDS. The data used for the analysis described in this manuscript and this Fig. were obtained from the GTEx Portal on 02/05/19. We indicated the Sigma Aldrich reference of each shRNA targeting MPV17 transcripts (sh128669, sh131201, sh131038, sh127649 and sh129921) above its targeted site. Each box represents an exon in MPV17 transcript isoforms. The darker the purple, the more abundant the transcript (as referred by Log10 (TPM)). The 3’UTR is located on the left of the image, and the 5’UTR on the right. We added an asterisk (*) that indicates both shRNAs providing the reduced proliferation phenotype, while ø indicates both shRNAs leading to an unchanged proliferation rate.
