# Supplementary material for: MPV17 does not control cancer cell proliferation
Source: PLoS One. 2020 Mar 10;15(3):e0229834. doi: 10.1371/journal.pone.0229834 (PMC7064194; doi:10.1371/journal.pone.0229834)
Supplement: S6 Fig — Protein immunostaining was performed with secondary antibodies coupled to infrared dyes (IRDye; green: 800nm/red: 700 nm). a: Full blot of the one presented in Figs 2A and 7A; b: Full blot of the one presented in Figs 4A and 7B (Anti-ATF4 from Santa Cruz); c: Full blot of the one presented in Fig 8A; d: Full blot of the one presented in Fig 4C (Anti-ATF4 from Cell Signaling); e: Full blot of the one presented in Fig 4B (Anti-ATF4 from Cell Signaling); f: Full blot of the one presented in Fig 5B; g: Full blot of the one presented in Fig 6B; h and i: Full blots of the ones presented in S3 Fig; j: Full blot of the one presented in Fig 6C (Anti-ATF4 from Santa Cruz). (DOCX) [file pone.0229834.s006.docx]

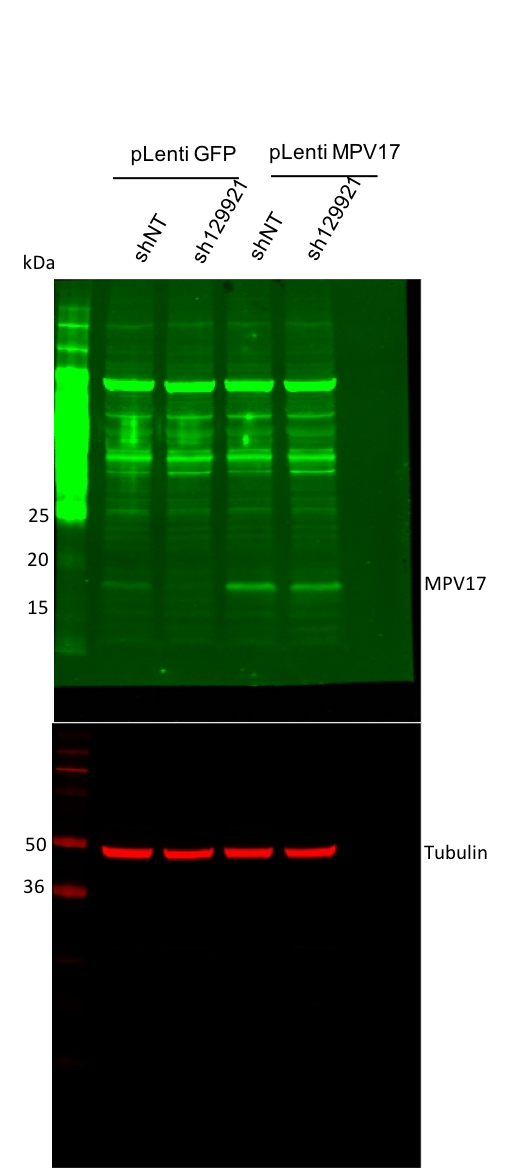

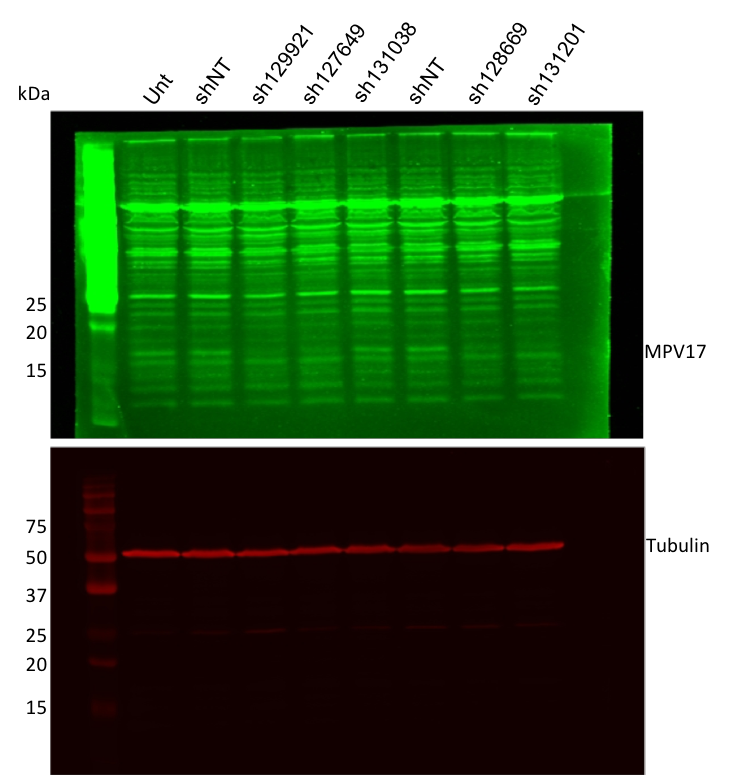

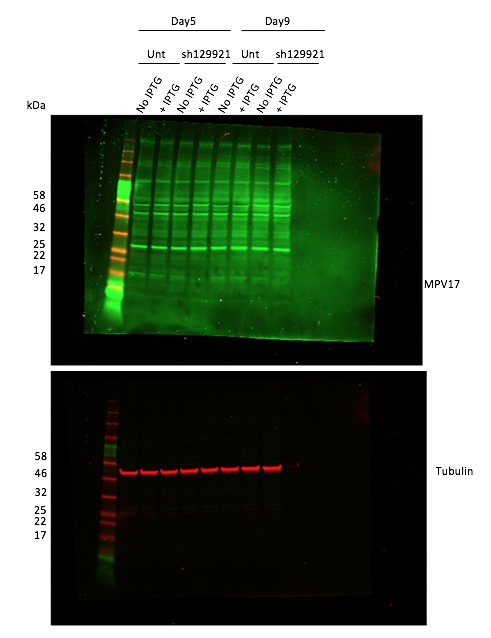

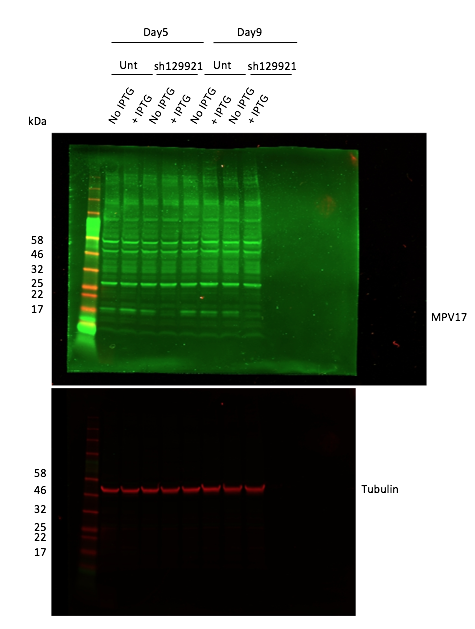

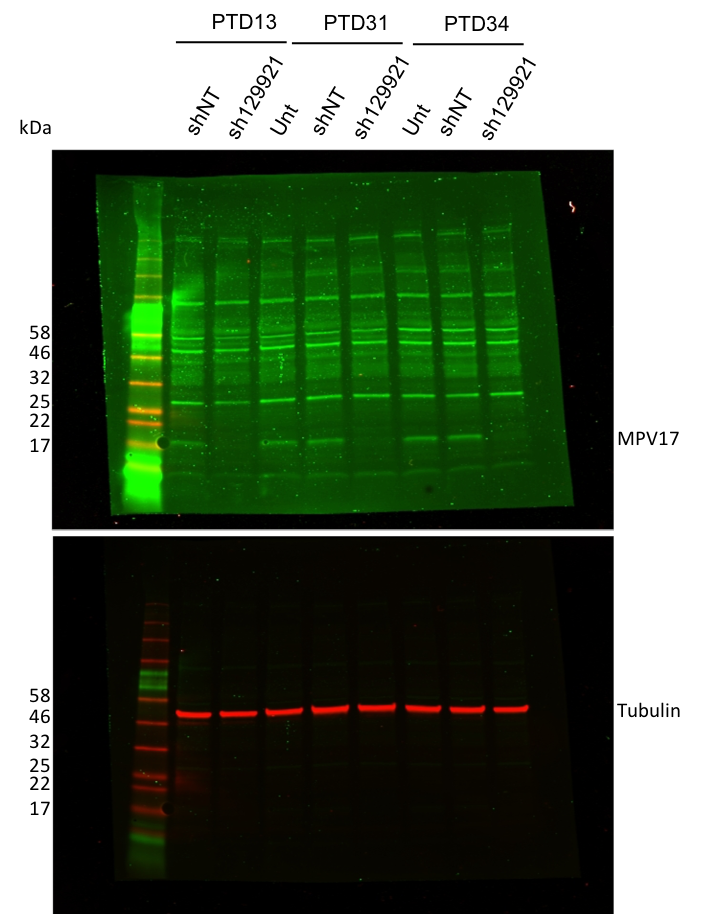

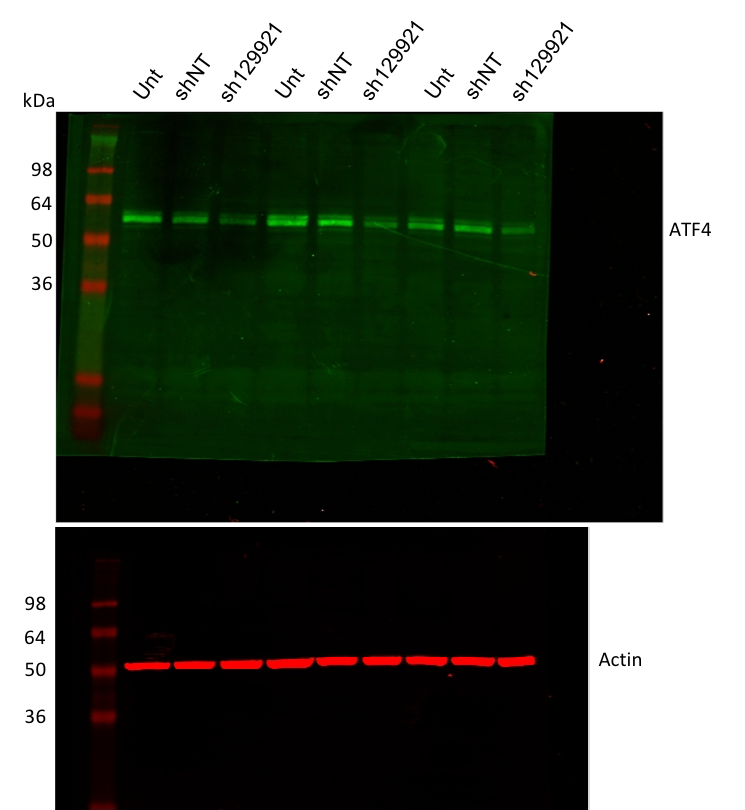

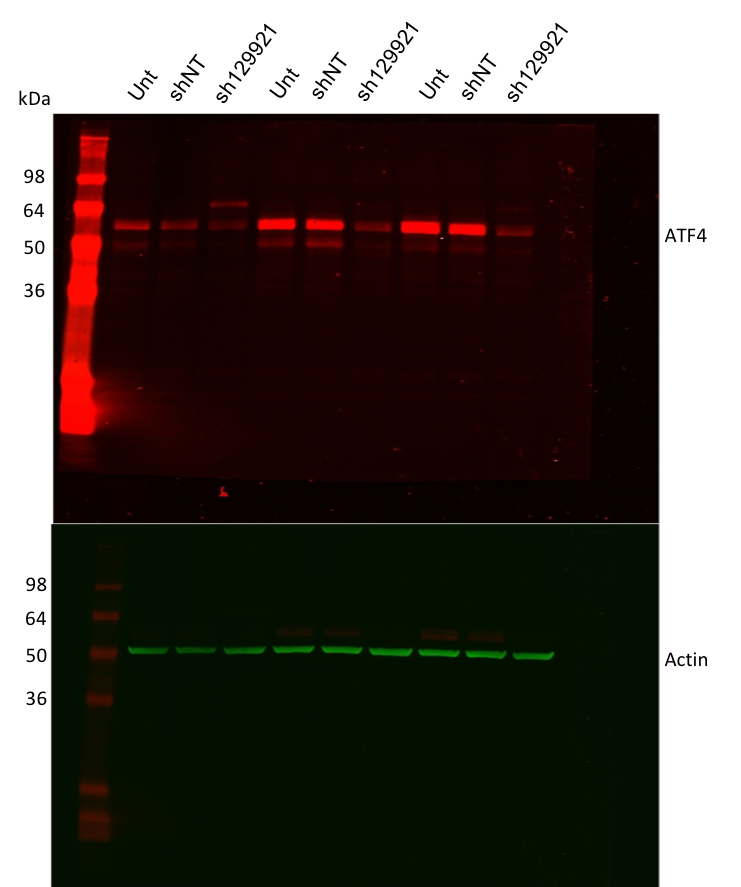

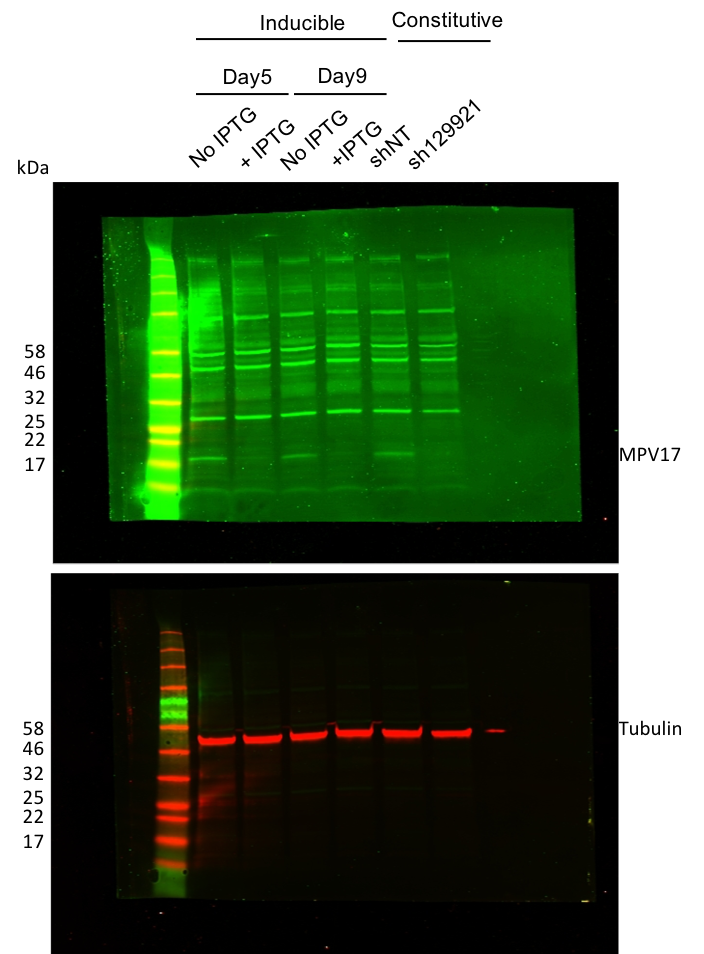


**S6 Fig. Full-length blots of the cropped blots presented in this work.** Protein immunostaining was performed with secondary antibodies coupled to infrared dyes (IRDye; green: 800nm/red: 700 nm).

a: Full blot of the one presented in Figs 2a and 7a; b: Full blot of the one presented in Figs 4a and 7b (Anti-ATF4 from Santa Cruz); c: Full blot of the one presented in Fig 8a; d: Full blot of the one presented in Fig 4c (Anti-ATF4 from Cell Signaling); e: Full blot of the one presented in Fig 4b (Anti-ATF4 from Cell Signaling); f: Full blot of the one presented in Fig 5b; g: Full blot of the one presented in Fig 6b; h and i: Full blots of the ones presented in S3 Fig; j: Full blot of the one presented in Fig 6c (Anti-ATF4 from Santa Cruz).

c

b

a

j

g

h

i

e

f

d
